# Chromones as Nonclassical Inhibitors of Carbonic Anhydrase IX and XII Isoforms: Probing Chromone‐Based Derivatives

**DOI:** 10.1002/ardp.70224

**Published:** 2026-03-16

**Authors:** Lisa Sequeira, Simona Distinto, Carlos Fernandes, Erica Sanna, Rita Meleddu, Marco Gaspari, Filippo Cottiglia, Alessia Onali, Andrea Angeli, Fernanda Borges, Eugenio Uriarte, Stefano Alcaro, Claudiu T. Supuran, Elias Maccioni

**Affiliations:** ^1^ MedInUP, Department of Biomedicine, Faculty of Medicine University of Porto Porto Portugal; ^2^ Department of Life and Environmental Sciences University of Cagliari Monserrato Italy; ^3^ Research Centre for Advanced Biochemistry and Molecular Biology, Department of Experimental and Clinical Medicine, “Magna Græcia” University of Catanzaro Catanzaro Italy; ^4^ Department NEUROFARBA, Section of Pharmaceutical Sciences University of Florence Florence Italy; ^5^ Department of Chemistry and Biochemistry, Faculty of Sciences University of Porto Porto Portugal; ^6^ Department of Organic Chemistry, Faculty of Pharmacy University of Santiago de Compostela Santiago de Compostela Spain; ^7^ Department of Health Sciences, “Magna Græcia” University of Catanzaro Catanzaro Italy

**Keywords:** cancer, carbonic anhydrase, docking, isoform expression, nonclassical inhibitors

## Abstract

A small library of differently substituted chromones was successfully synthesized and structurally characterized. All compounds were evaluated for their inhibitory potency and selectivity toward human cancer‐associated carbonic anhydrase isoforms IX and XII, as well as the off‐target isoforms I and II. Compounds **4a**, **4g**, **4j**, and **4k** selectively inhibited cancer‐associated isoforms IX and XII, with no activity against the off‐target isozymes I and II. Among them, compound **4k** was the most potent and isozyme‐selective inhibitor, with *K*_i_ 0.31 µM for *h*CA IX and 0.24 µM for *h*CA XII. To estimate drug‐likeness, in silico ADMET predictions were performed, indicating that all compounds possess physicochemical and pharmacokinetic properties within the acceptable ranges. Molecular docking studies on the *h*CA IX isoform highlighted an optimal orientation within the binding pocket, with the chromene moiety positioned toward the zinc ion. In cellular assays **4a**, **4g**, **4j**, and **4k** selectively inhibited metabolic activity in HepG2 cells expressing *h*CA IX in normal conditions, whereas no activity was observed in Caco‐2 cells lacking *h*CA IX expression.

## Graphical abstract

Chromone‐based derivatives were synthesized and evaluated for selective inhibition of tumor‐associated carbonic anhydrases IX and XII. Structure–activity relationships, docking studies, and ADMET profiling revealed promising candidates with selective tumoral isoform inhibition, supporting their potential as anticancer leads.

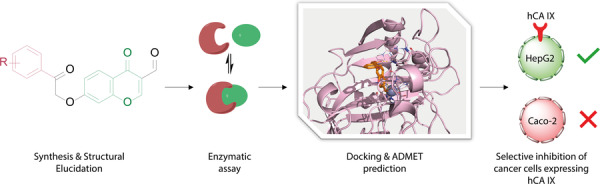

## 1 Introduction

Chromone (4*H*‐chromen‐4‐one, also known as 4*H*‐1‐benzopyran‐4‐one) is the scaffold of an important class of oxygen‐containing heterocyclic chemical entities, namely the flavonoid family [1, 2, 3]. Owing to their structural diversity, chromone derivatives are generally categorized into simple chromones and fused systems, including pyranochromones and furanochromones [3, 4].

**Figure 1:**
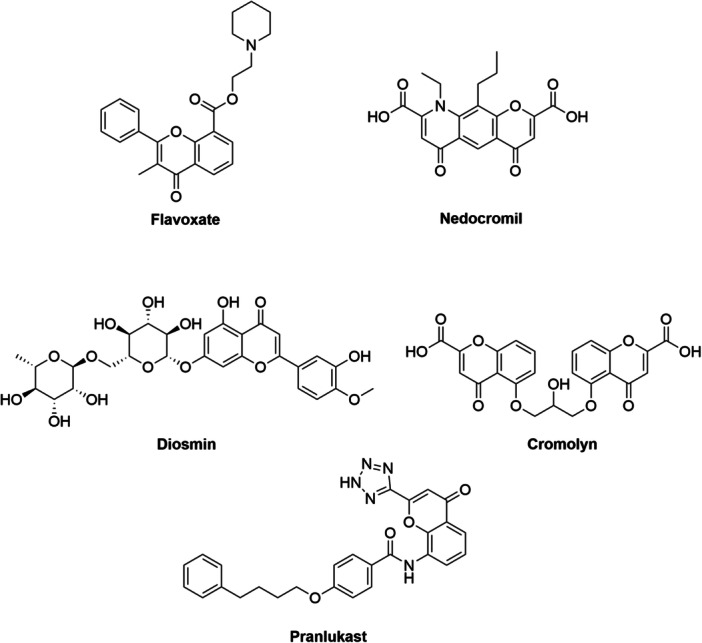
Examples of chromone‐based compounds used as pharmaceutical agents: Flavoxate, anticholinergic [37]; Nedocromil, antiasthmatic [38]; Diosmin, vasoprotective and venotonic agent [39]; Cromolyn, antiallergic and mast cell stabilizer [40]; Pranlukast, antiasthmatic and leukotriene receptor antagonist [41].

This moiety, widely represented in the plant kingdom, has attracted significant attention due to its wide range of properties [3]. Numerous pharmacological activities have been attributed to simple chromones and their analogs, such as antibacterial, antifungal [5], anticancer [4, 5, 6], antioxidant [7], anti‐HIV [8], immunostimulatory [9], wound‐healing [10], analgesic, and anti‐inflammatory [11] activities. Because of this versatility, the chromone nucleus is recognized as a privileged structural motif in medicinal chemistry, frequently incorporated into the design of new bioactive molecules and several marketed drugs [1, 2, 3, 4]. Representative examples of therapeutic agents containing the chromone scaffold are illustrated in Figure 1 [1]. Most investigations on chromone derivatives for cancer therapy have focused on identifying novel kinase inhibitors [12]. However, other molecular targets, such as carbonic anhydrases (CAs), have also been explored [13, 14, 15]. CAs are zinc metalloenzymes distributed in all living organisms that catalyze the reversible hydration reaction of carbon dioxide into bicarbonate and a proton [16, 17, 18]. Eight evolutionarily distinct CA families are currently recognized: the α‐CAs (present in vertebrates, plants, Bacteria, Archaea, cyanobacteria marine diatoms, protozoa, and some filamentous ascomycetes) [19], the β‐CAs (predominantly in Bacteria, Archaea, cyanobacteria, protozoa and filamentous ascomycetes) [20], the γ‐CAs (mainly in Bacteria, Archaea and cyanobacteria) [21], the δ‐CAs (recognized in some marine diatoms and the fungal kingdom) [22], the ζ‐CAs (encoded by marine diatoms), the η‐CAs (detected in protozoa), the θ‐CAs (contained in marine diatoms) [23], and the ι‐CAs (present in Bacteria, Archaea, cyanobacteria and marine diatoms) [24]. The α‐class is the only class present in mammals and comprises sixteen isoforms differing in cellular localization and catalytic properties [25, 26, 27]. Among these, CA I, II, III, VII, VIII, X, XI and XIII are expressed in the cytoplasm [28]; CA IX, XII, and XIV are membrane bound forms [28, 29]; CA IV and XV the latter not expressed in humans [30] are glycosylphosphatidylinositol (GPI)‐anchored membrane forms [28, 31]; CA VA and VB are mitochondrial forms [32] and CA VI is a saliva‐secreted form [33]. Three of the cytosolic isoforms (VIII, X, and XI), known as CA‐related proteins (CARPs), are catalytically inactive because they lack one or more histidine residues that coordinate the zinc ion in the active site. In contrast, the transmembrane isoforms are highly active enzymes and glycoproteins [34, 35, 36].

Tumor‐associated CA IX and XII have been consistently validated as targets of disease progression in many solid tumors. Experimental evidence showed a strong link between pH regulation and tumor cell proliferation and survival [42]. These two transmembrane isoforms are actively involved in carbon dioxide metabolism and consequently play a role in pH control and tumor progression. Both enzymes are subject to several post‐translational modifications, particularly glycosylation, which influences their folding, stability, localization, and catalytic activity. Their expression is largely controlled by the hypoxia‐inducible factor (HIF) pathway, and their upregulation in hypoxic tumors has established them as promising diagnostic markers and therapeutic targets [43, 44, 45]. Consequently, research efforts in recent decades have focused on these transmembrane enzymes [43, 46, 47]. The membrane‐bound CA isoforms CA IX and CA XII are the primary CA isoforms expressed in cancer [43, 48]. Particularly, CA IX is almost exclusively expressed in a broad range of tumors, representing a reliable indicator of malignant lesions [43, 49, 50, 51]. The biological relevance of CA XII has been less thoroughly investigated, likely because, although it is overexpressed in many cancers, it is also found in several normal tissues [52, 53, 54]. Current therapeutic approaches targeting tumor‐associated CAs mainly rely on two strategies: the generation of monoclonal antibodies and the design of selective small‐molecule inhibitors directed at CA IX and CA XII [27, 55].

**Scheme 1:**
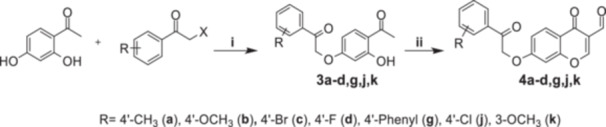
Synthetic pathway to obtain compounds **4a–d**, **4g**, **4j**, and **4k**. Reagents and conditions: (i) 2′,4′‐dihydroxyacetophenone, α‐haloketone, acetone, reflux (3–6 h); POCl_3_, DMF, −10°C (1 h), room temperature (3–6 days).

Building on our group's ongoing research in the field of CAs and anticancer agents [16, 56, 57, 58, 59, 60, 61, 62], we designed and synthesized a new series of 4H‐chromen derivatives to evaluate their activity and selectivity against *h*CA IX and XII, in comparison to the off‐target isoforms *h*CA I and II. All the synthesized chromone‐based derivatives (**4a–d**, **4g**, **4j**, and **4k**) have an aldehyde group in Position 3 and a 2‐oxo‐2‐arylethoxy group in Position 7 with a differently substituted aromatic ring (Scheme 1). In addition to the biological screening against CAs, the cytotoxic profile of the new compounds was also evaluated in human hepatocarcinoma (HepG2) and adenocarcinoma (Caco‐2) cell lines by measuring, after 24 h of treatment, the cellular metabolic activity and mass through the resazurin reduction method and sulforhodamine‐B assays, respectively.

## 2 Results and Discussion

### 2.1 Chemistry

The synthetic procedure used to obtain compounds **4a–d**, **4g**, **4j**, and **4k** is represented in Scheme 1. Starting **3a–d**, **3g**, **3j**, **3k** intermediates were synthesized by Williamson reaction (reactional Step i) between 2,4‐dihydroxyphenylethan‐1‐one and an α‐haloketone with the desired substitution. Compounds **3a–d**, **3g**, **3j**, and **3k** were submitted to a POCl_3_‐induced cyclization (reactional Step ii) to obtain the corresponding chromone‐based derivatives (compounds **4a–d**, **4g**, **4j**, and **4k**). All final compounds were characterized using analytical methods, including ^1^H‐NMR, ^13^C‐NMR, and high‐resolution mass spectrometry (HRMS), before being submitted to biological evaluation.

### 2.2 Biological Activity

The synthesized chromone‐based derivatives **4a–d**, **4g**, **4j**, and **4k** were evaluated against *h*CA I, II, IX, and XII isoforms to assess their inhibitory activity and selectivity toward the different isozymes. Acetazolamide (AAZ), a known potent but unselective inhibitor of *h*CA, was used as a reference standard. The results are summarized in Table 1.

**Table 1:** Inhibition data toward *h*CA I, II, IX, and XII of compounds 4a–d, 4g, 4j, and 4k.

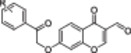					
Compound	R	*K*_i_ (UM)			
		*h*CA I	*h*CA II	*h*CA IX	*h*CA XII
**4a**	4′‐CH_3_	> 100	> 100	0.44	0.33
**4b**	4′‐OCH_3_	> 100	> 100	> 100	> 100
**4c**	4′‐Br	> 100	> 100	> 100	> 100
**4d**	4′‐F	> 100	> 100	> 100	> 100
**4g**	4′‐C_6_H_5_	> 100	> 100	0.86	0.69
**4j**	4′‐Cl	> 100	> 100	0.42	0.28
**4k**	3′‐OCH_3_	> 100	> 100	0.31	0.24
**AAZ**	//	0.250	0.0125	0.026	0.0057

Accordingly, although all the active compounds exhibited lower potency than the reference compound AAZ, some important conclusions regarding selectivity must be highlighted. None of the compounds showed activity against CA I or CA II at concentrations up to 100 µM (the highest tested concentration), indicating excellent selectivity for tumor‐associated isoforms. Substitution at Position 7 of the chromone scaffold with a 4′‐methoxyphenyl, 4′‐bromophenyl, or 4′‐fluorophenyl moiety (in compounds **4b**, **4c**, and **4d**, respectively) resulted in complete loss of activity toward all isoforms. In contrast, the other compounds (**4a**, **4g**, **4j**, and **4k**) exhibited similar activity at low micromolar concentrations against CA IX and CA XII. Among them, **4k**, bearing a 3′‐methoxyphenyl group at Position 7 of the chromone scaffold, showed the most relevant inhibitory activity and selectivity toward CA IX and CA XII, with *K*_i_ values of 0.31 and 0.24 µM, respectively [59, 62].

### 2.3 Molecular Docking

To gain insight into the binding mode and acquire structural information that could support future compound design and optimization, the two derivatives showing the highest potency and selectivity toward hCA IX and XII isoforms were selected. Their binding modes were investigated using a validated docking protocol [58, 62, 63].

**Figure 2:**
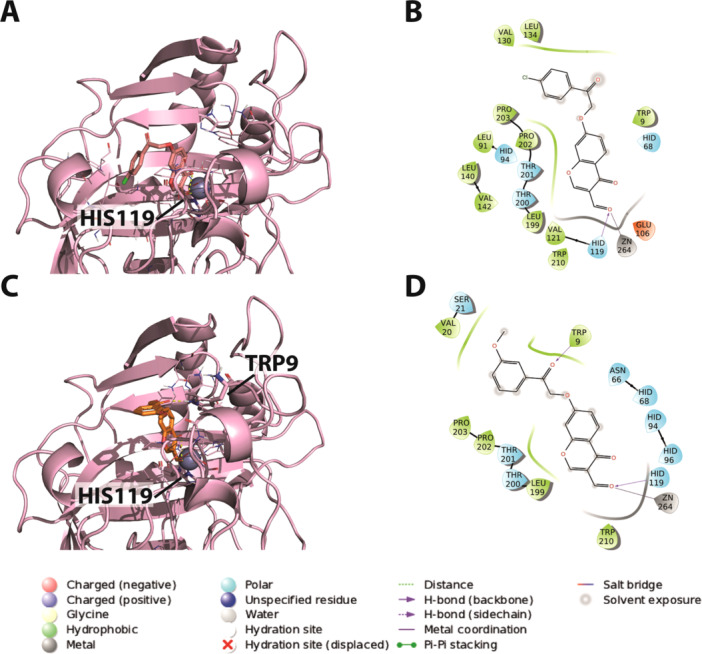
Predicted binding modes of the most potent compounds (**4j** and **4k**) with hCA IX based on molecular docking. (A) 3D depiction of **4j** and its respective interactions with CA IX residues. (B) 2D depiction of interactions. (C) 3D depiction of **4k** and its respective interactions with CA IX residues. (D) 2D depiction of interactions.

The putative binding modes of **4j** and **4k**, obtained through docking experiments on the hCA IX isoform, are depicted in Figure 2. The results reveal their potential interactions within the enzyme's active site. Specifically, both compounds exhibit a binding orientation in which the chromene ring is directed toward the zinc ion, which is known to coordinate with various ligands in the active site of CA enzymes. Additionally, a hydrogen bond interaction with His119, a crucial residue in the active site, is observed. The flexible benzyl tail of the compounds interacts with the entrance cavity of the enzyme.

Despite these insights, the precise molecular basis underlying the enhanced potency and selectivity of derivatives **4j** and **4k** compared to their inactive counterparts remains to be fully elucidated. The data suggest that an optimal combination of steric and electronic effects may be critical for achieving high affinity and selectivity toward *h*CA IX and XII.

**Figure 3:**
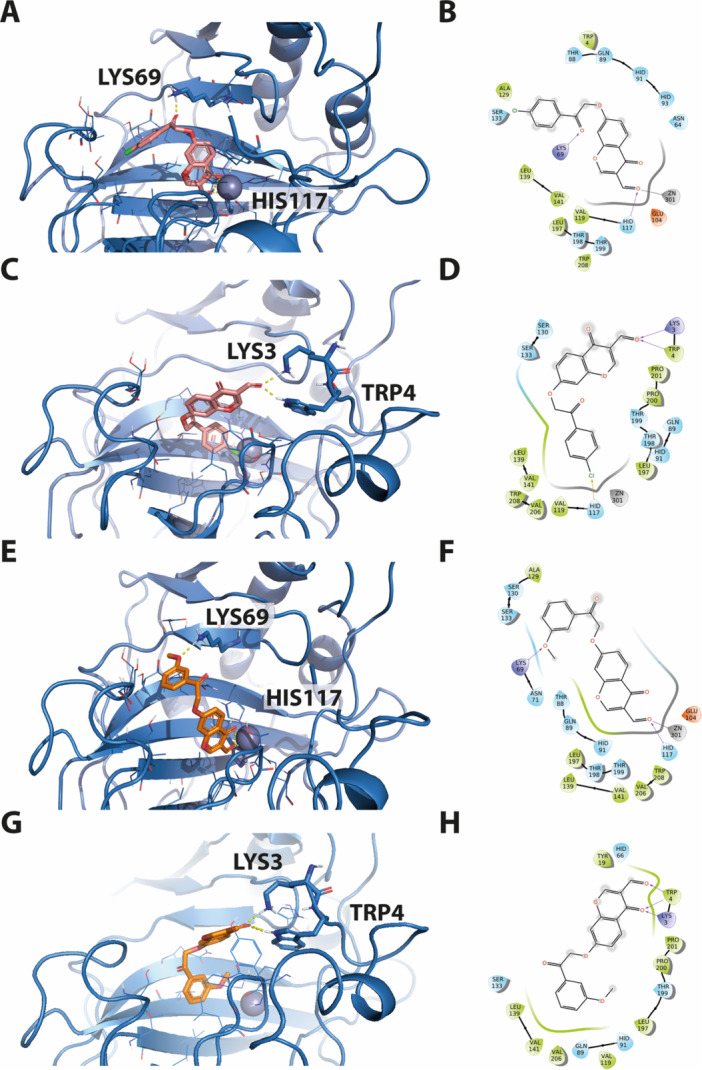
Putative binding modes of the most potent compounds (**4j** and **4k**) with CA XII as predicted by molecular docking. (A) 3D representation of compound **4j** and its interactions with CA XII residues. (B) Corresponding 2D interaction diagram of **4j**. (C) Alternative 3D binding orientation of **4j** within the CA XII active site. (D) 2D interaction diagram for the alternative binding mode of **4j**. (E) 3D representation of compound **4k** and its interactions with CA XII residues. (F) Corresponding 2D interaction diagram of **4k**. (G) Alternative 3D binding orientation of **4k** within the CA XII active site. (H) 2D interaction diagram for the alternative binding mode of **4k**.

To explore these structure–activity trends, docking experiments were performed on the *h*CA XII isoform for compounds **4j** and **4k**. Notably, the simulations revealed two distinct binding orientations differing from those observed in the *h*CA IX isoform, as shown in Figure 3. In the first orientation, the chromene ring is directed toward the zinc ion, similarly to its orientation in the IX isoform. In the second alternative pose, the substituted phenyl ring faces the zinc ion, while hydrogen bonding interactions with Lys3 and Trp4 contribute to the stabilization of the chromone moiety.

These findings underscore the flexibility of ligand binding and suggest that multiple binding modes may contribute to isoform selectivity. Notably, experimental studies have shown that ligands can adopt more than one well‐defined position within crystal structures, often modeled as alternate conformations visible in electron density maps [64, 65]. Similar dual binding conformations have also been reported in docking studies [66, 67], supporting the reliability of these in silico findings and indicating that alternate docking poses may reflect real coexisting states in this protein–ligand complex. However, further investigation may be needed to fully define the structural determinants of binding and activity of this compound series.

Structural biology techniques such as x‐ray crystallography, complemented by the design and synthesis of systematically varied analogs and derivatives, could provide deeper insights into the specific interactions between these compounds and the enzyme. Such investigations are crucial for unraveling the molecular basis of their activity and guiding future optimization efforts. Nevertheless, the presented results support the potential of chromone‐based scaffold as a promising candidate for the selective inhibition of the tumor‐associated isoforms *h*CA IX and XII.

### 2.4 Drug‐Like Properties

Given the importance of early evaluation of drug‐like properties, all the compounds under investigation were submitted to theoretical ADMET (absorption, distribution, metabolism, excretion, and toxicity) calculations using the QikProp Schrodinger software [68] (Tables S1–S4). Overall, chromone‐based derivatives showed a good drug‐like profile.

Furthermore, the drug‐like properties of the most active compounds were evaluated using biomimetic HPLC, following the methodology described by Valko et al. [69]. This approach enabled the determination of the chromatographic hydrophobicity index (CHI), the chromatographic hydrophobicity index using immobilized artificial membrane (CHI(IAM)), and percentage of binding to human serum albumin (%HSA) (Table 2), based on linear regression correlations between retention times and known CHI, CHI(IAM) and %HSA values from a reference set of compounds tested in a mixed solution. Additionally, LogP, Kpcell, Vdu, and DEmax values (Table 2) were calculated according to the literature [70].

**Table 2:** Lipophilicity (CHI and LogP), phospholipid binding (CHI IAM), estimated cell partition (Kpcell), human albumin binding (%HSA), the unbound volume of distribution (Vdu), and drug efficiency (DEmax)^a^ of the selected chromone‐based derivatives.

Compound	CHI	LogP	CHI(IAM)	K(Pcell)	%HSA	Vdu	DEmax (%)
**4a**	73.68	2.36	50.18	12.62	74.45	4.37	22.88
**4g**	87.64	3.02	48.33	10.18	78.41	4.39	22.77
**4j**	76.73	2.51	50.53	13.14	74.38	4.43	22.58
**4k**	68.82	2.13	37.29	2.81	72.10	2.65	37.71

^a^ Values obtained by the extrapolation of linear regression or by calculations considering the equations in the literature.

The data showed that the compounds exhibited LogP values in the range of 2–3, consistent with a high predicted permeability across biological barriers such as the intestinal and blood‐brain barriers [71]. Furthermore, the LogP values obtained correlate with the type of aromatic ring substituents, allowing a ranking of hydrophobicity: phenyl group > chloride group > methyl group > methoxyl group.

Using an IAM chromatographic column, which mimics the phospholipidic bilayer of biological membranes, the interaction of the compounds with cellular membranes, especially phospholipidic membranes, was estimated. From the CHI(IAM) values obtained through linear regression, the K(pcell) parameter was calculated, which estimates the concentration ratio of the compound inside versus outside the cell. According to the values presented in Table 2, compounds **4a**, **4g**, and **4j** showed a higher capacity for cellular internalization compared to **4k**.

The binding of drug substances to plasma proteins, such as human serum albumin [72], α‐acid glycoprotein, and lipoproteins, is closely linked to their effective concentration at the target site [73]. All the tested compounds exhibited an estimated HAS binding capacity of approximately 72%–78%, indicating that in vivo, around 20%–30% of the compound would remain unbound in the plasma. These data are critical for estimating the unbound volume of distribution (Vdu) and the maximum drug efficiency (DEmax). The Vdu is determined by the compounds binding to phospholipid membranes and HAS, and it is proportional to the administered dose divided by the free plasma concentration [70]. In the absence of significant active transport or permeability barriers, the reciprocal of the Vdu corresponds to DEmax, which reflects the free biophase concentration, that is, the unbound concentration of the compound at its site of action relative to the administered dose. This makes it a key parameter during the lead optimization process.

Among the compounds tested, **4k** presents the highest free drug concentration and, accordingly, the highest DEmax value (37.71), suggesting a potentially greater therapeutic efficacy in in vivo models.

### 2.5 Cytotoxicity Profile

**Figure 4:**
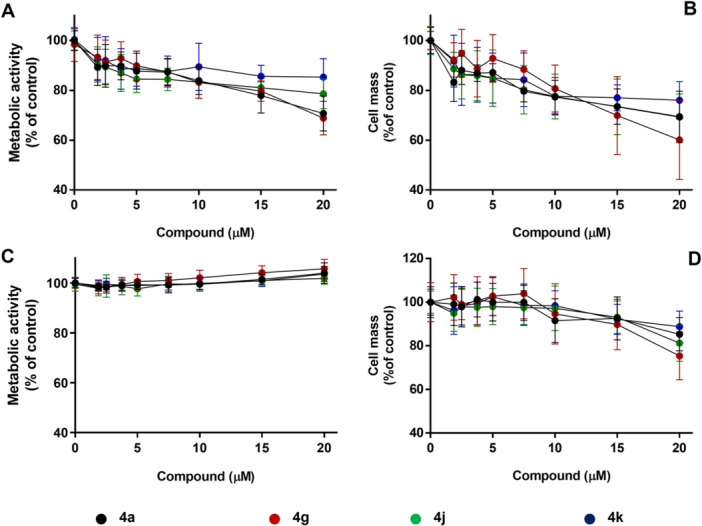
Evaluation of cell viability using HepG2 (A and B) and Caco‐2 (C and D) cell lines after 24 h of cellular treatment with compounds **4a**, **4g**, **4j**, and **4k** (0–20 μM), followed by the measurement of metabolic activity (A and C) and cell mass (B and D) using resazurin and SRB assays. The results are presented as mean ± SD of triplicates from at least three independent experiments (*n* ≥ 9).

The cytotoxic profile of the compounds **4a**, **4g**, **4j**, and **4k** at a range of different concentrations (0–20 μM) was assessed in hepatocarcinoma (HepG2) and adenocarcinoma (Caco‐2) cell lines after 24 h of exposure using the resazurin reduction to assess metabolic activity and sulforhodamine B (SRB) uptake assays, to assess cell mass, as endpoints to measure cell viability. The two cell lines were selected based on their differential CA IX expression profiles under normoxic conditions to explore the cytotoxic effects of compounds **4a**, **4g**, **4j**, and **4k** with or without expression of CAIX in normal culture conditions. Several works described that CAIX is only expressed in Caco‐2 cells under hypoxic conditions [74, 75], whereas HepG2 cells express CA IX under normal conditions [76]. The results are shown in Figure 4.

After 24 h of treatment, all compounds exhibited cytotoxic effects on HepG2 cells in a concentration‐dependent manner, as measured by both metabolic activity and cell mass endpoints (Figures 4A,B). In this cell line, at the highest concentration tested, a cytotoxic ranking was established as follows: **4g** > **4a** ≈ **4j** > **4k**, with cell viability values in the range of 60.0%–85.3%. These findings are supported by the CHI(IAM) results (Table 2), which suggest a higher lipophilicity profile and cellular internalization for compounds **4a**, **4g**, and **4k**. A trend correlation between these two ADMET parameters and the metabolic activity data (Figure 4) was observed in HepG2 cells. Hence, a better membrane permeability correlates with increased cytotoxic effects in CA IX‐expressing cells.

In contrast, the metabolic activity of Caco‐2 cells was not affected after 24 h of treatment with the compounds under study. Despite a slight depletion in Caco‐2 cell mass (Figure 4D), the overall cytotoxicity profile differed notably from that observed in HepG2 cells (Figure 4B), which could potentially be associated with CA IX inhibition, reinforcing the hypothesis that the antiproliferative effects of these chromone derivatives are at least partly CA IX‐dependent.

## 3 Conclusions

Although chromone‐based derivatives are weaker inhibitors of the four hCA isoforms compared to the reference AAZ, most of them are selective toward hCA IX and XII. Substitution with methyl, phenyl, or chlorine groups at Position 4, and a methoxy group at Position 3 of the aromatic ring, enhanced both activity and selectivity toward hCA IX and XII isozymes. Preliminary in silico studies indicate that all chromone‐based derivatives exhibit favorable drug‐like properties. Among them, compounds **4a**, **4g**, **4j**, and **4k** significantly reduced HepG2 cell viability after 24 h of treatment, and a cytotoxic ranking was established as follows: **4g** > **4a** ≈ **4j** > **4k**. These effects were correlated with their lipophilicity and membrane permeability, as evaluated by biomimetic chromatographic experiments. Notably, no cytotoxicity was observed in terms of metabolic activity in Caco‐2 cells. These findings support the further optimization of chromone‐based derivatives to improve both activity and isozyme selectivity.

## 4 Experimental

### 4.1 Chemistry

#### 4.1.1 General

Starting materials, reagents, and solvents were obtained from commercial suppliers and were used without any further purification. NMR data were acquired on a Bruker Avance III 400 NMR spectrometer at room temperature, operating at 400.15 MHz for 1H and 100.62 MHz for 13C and DEPT135 (Distortionless Enhancement by Polarization Transfer). Tetramethylsilane (TMS) was used as an internal reference; chemical shifts (*δ*) were expressed in ppm, and coupling constants (J) were given in Hz. DEPT135 values were included in ^13^C NMR data (underline values). Mass spectra were carried out on a Varian 320‐MS (EI) or Bruker Microtof (ESI) apparatus; the data were reported as *m/z* (% of relative intensity of the most important fragments). NMR spectra were registered on a Bruker AMX (400 MHz) spectrometer. TLC was performed using silica gel plates (Merck F 254), and spots were visualized by UV light. Mass spectra were acquired on an Orbitrap Exploris mass spectrometer (Thermo Fisher Scientific, Germany). Compounds were initially dissolved in dimethylsulfoxide (DMSO) at 5 mg/mL concentration. Stock solutions were then diluted 50‐fold in acetonitrile and further diluted 10‐fold in 70% acetonitrile containing 0.1% of formic acid. Solutions were directly infused into the mass spectrometer at 5 µL/min. Mass spectra were acquired in positive ion mode (3200 V). Ion transfer tube temperature was 320°C, whereas S‐lens value was 20 units. Full MS spectra were acquired at a resolution of 240,000, in the *m/z* range 180–1000 (Table S5). Melting points were determined by the capillary method on a Stuart Scientific melting point apparatus and are uncorrected. Supporting Information (SI) contains NMR and HRMS data.

#### 4.1.2 General Procedure for the Synthesis of 3a–d, 3g, 3j, and 3k Intermediates

To a mixture of 2′,4′‐dihydroxyacetophenone (1 mmol) and acetone (20 mL) was added K_2_CO_3_ (2.5 mmol). This mixture was kept stirring at 40°C for 30 min. The appropriate α‐haloketone (1.1 mmol) was added. The reaction mixture was heated to reflux and stirred. Upon completion, the mixture was poured into a solution of H_2_SO_4_ 0.5 M (100 mL), and the resulting precipitate was filtered. The crude product was purified by silica gel flash column chromatography; the resulting fractions containing the desired compound were collected, and the solvent was evaporated to dryness under vacuum and recrystallized from CH_2_Cl_2_/n‐hexane to obtain the pure compound. The procedure was adapted from the literature [77].

2‐(4‐Acetyl‐3‐hydroxyphenoxy)‐1‐(4‐methylphenyl)ethan‐1‐one (**3a**): Yield: 72.6%. 2‐Bromo‐4′‐methylacetophenone as α‐haloketone. Purified by silica gel flash column chromatography with CH_2_Cl_2_. ^1^H NMR (400 MHz, CDCl_3_): *δ* = 2.44 (*s*, 3H, CH_3_), 2.55 (*s*, 3H, COCH_3_), 5.30 (*s*, 2H, CH_2_), 6.39 (*d*, *J* = 2.5 Hz, 1H, H3), 6.54 (*dd*, *J* = 8.9, 2.6 Hz, 1H, H5), 7.29–7.32 (*m*, 2H, 2 × H(Ar)), 7.65 (*d*, *J* = 8.9 Hz, 1H, H6), 7.86–7.88 (*m*, 2H, 2 × H(Ar′)), and 12.68 (*s*, 1H, OH). ^13^C NMR (100 MHz, CDCl_3_): *δ* = 21.80 (CH_3_), 26.27 (COCH_3_), 70.27 (CH_2_), 101.84 (C(Ar)), 107.92 (C(Ar)), 114.54 (C(Ar)), 128.12 (2 × C(Ar′)), 129.65 (2 × C(Ar′)), 131.75 (C(Ar)), 132.52 (C(Ar)), 145.22 (C(Ar)), 164.37 (C(Ar)), 165.05 (C(Ar)), 192.58 (CO), and 202.70 (COCH_3_).

2‐(4‐Acetyl‐3‐hydroxyphenoxy)‐1‐(4‐methoxyphenyl)ethan‐1‐one (**3b**): Yield: 97.1%. 2‐Bromo‐4′‐methoxyacetophenone as α‐haloketone. ^1^H NMR (400 MHz, CDCl_3_): *δ* = 2.55 (*s*, 3H, CH_3_), 3.89 (*s*, 3H, OCH_3_), 5.27 (*s*, 2H, CH_2_), 6.39 (*d*, *J* = 2.5 Hz, 1H, H3), 6.53 (*dd*, *J* = 8.9, 2.6 Hz, 1H, H5), 6.96–6.99 (*m*, 2H, 2 × H(Ar′)), 7.65 (*d*, *J* = 8.9 Hz, 1H, H6), 7.94–7.98 (*m*, 2H, 2 × H(Ar′)), and 12.68 (*s*, 1H, OH). ^13^C NMR (100 MHz, CDCl_3_): *δ* = 26.27 (CH_3_), 55.58 (COCH_3_), 70.22 (CH_2_), 101.87 (C(Ar)), 107.89 (C(Ar)), 114.17 (2 × C(Ar′)), 114.53 (C(Ar)), 127.27 (C(Ar)), 130.42 (2 × C(Ar′)), 132.52 (C(Ar)), 164.28 (C(Ar)), 164.42 (C(Ar)), 165.05 (C(Ar)), 191.49 (CO), and 202.69 (COCH_3_).

2‐(4‐Acetyl‐3‐hydroxyphenoxy)‐1‐(4‐bromophenyl)ethan‐1‐one (**3c**): Yield: 78.9%. 2,4′‐Dibromoacetophenone as α‐haloketone. Purified by silica gel flash column chromatography with CH_2_Cl_2_. ^1^H NMR (400 MHz, CDCl_3_): *δ* = 2.58 (*s*, 3H, COCH_3_), 5.29 (*s*, 2H, CH_2_), 6.40 (*d*, *J* = 2.5 Hz, 1H, H3), 6.54 (*dd*, *J* = 8.9, 2.6 Hz, 1H, H5), 7.66–7.69 (*m*, 3H, 3 × H(Ar)), 7.85–7.88 (*m*, 2H, 2 × H(Ar′)), and 12.70 (*s*, 1H, OH). ^13^C NMR (100 MHz, CDCl_3_): *δ* = 26.30 (COCH_3_), 70.35 (CH_2_), 101.81 (C(Ar)), 107.81 (C(Ar)), 114.68 (C(Ar)), 129.50 (C(Ar)), 129.59 (2 × C(Ar′)), 132.34 (2 × C(Ar′)), 132.60 (C(Ar)), 132.92 (C(Ar)), 164.04 (C(Ar)), 165.03 (C(Ar)), 192.39 (CO), and 202.74 (COCH_3_).

2‐(4‐Acetyl‐3‐hydroxyphenoxy)‐1‐(4‐fluorophenyl)ethan‐1‐one (**3d**): Yield 85.1%. 2‐Bromo‐4′‐fluoroacetophenone as α‐haloketone. Purified by silica gel flash column chromatography with CH_2_Cl_2_. ^1^H NMR (400 MHz, CDCl_3_): *δ* = 2.58 (*s*, 3H, COCH_3_), 5.30 (*s*, 2H, CH_2_), 6.40 (*d*, *J* = 2.6 Hz, 1H, H3), 6.55 (*dd*, *J* = 8.9, 2.6 Hz, 1H, H5), 7.18–7.24 (*m*, 2H, 2 × H(Ar′)), 7.68 (*d*, *J* = 8.9 Hz, 1H, H6), 8.01–8.06 (*m*, 2H, 2 × H(Ar′)), and 12.70 (*s*, 1H, OH). ^13^C NMR (100 MHz, CDCl_3_): *δ* = 26.28 (COCH_3_), 70.33 (CH_2_), 101.81 (C(Ar)), 107.83 (C(Ar)), 114.65 (C(Ar)), 116.23 (d, *J*_CF_ = 22.0 Hz, C3′, C5′), 130.68 (d, *J*_CF_ = 3.1 Hz, C1′), 130.88 (d, *J*_CF_ = 9.5 Hz, C2′, C6′), 132.58 (C(Ar)), 164.12 (C(Ar)), 165.01 (C(Ar)), 166.30 (d, *J*_CF_ = 254.0 Hz, C4′), 191.69 (CO), and 202.73 (COCH_3_).

2‐(4‐Acetyl‐3‐hydroxyphenoxy)‐1‐(4‐phenylphenyl)ethan‐1‐one (**3g**): Yield: 50.0%. 2‐Bromo‐1‐(4‐phenylphenyl)ethan‐1‐one as α‐haloketone. Purified by silica gel flash column chromatography with CH_2_Cl_2_. ^1^H NMR (400 MHz, CDCl_3_): *δ* = 2.56 (*s*, 3H, OCH_3_), 5.35 (*s*, 2H, CH_2_), 6.42 (*d*, *J* = 2.5 Hz, 1H, H3), 6.56 (*dd*, J = 8.9, 2.6 Hz, 1H, H5), 7.40–7.45 (*m*, 1H, H(Ar)), 7.45–7.51 (*m*, 2H, 2 × H(Ar)), 7.61–7.66 (*m*, 3H, 3 × H(Ar)), 7.71–7.76 (*m*, 1H, H(Ar)), 8.02–8.08 (*m*, 2H, 2 × H(Ar)), and 12.69 (*s*, 1H, OH). ^13^C NMR (100 MHz, CDCl_3_): *δ* = 26.28 (COCH_3_), 70.41 (CH_2_), 101.87 (C(Ar)), 107.91 (C(Ar)), 114.60 (C(Ar)), 127.31 (2 × C(Ar)), 127.57 (2 × C(Ar)), 128.54 (C(Ar)), 128.65 (2 × C(Ar)), 129.05 (2 × C(Ar)), 132.56 (C(Ar)), 132.88 (C(Ar)), 139.56 (C(Ar)), 146.89 (C(Ar)), 164.31 (C(Ar)), 165.07 (C(Ar)), 192.63 (CO), and 202.71 (COCH_3_).

2‐(4‐Acetyl‐3‐hydroxyphenoxy)‐1‐(4‐chlorophenyl)ethan‐1‐one (**3j**): Yield: 65.3%. 2‐Bromo‐4′‐chloroacetophenone as α‐haloketone. Purified by silica gel flash column chromatography with CH_2_Cl_2_. ^1^H NMR (400 MHz, CDCl_3_): *δ* = 2.58 (*s*, 3H, COCH_3_), 5.29 (*s*, 2H, CH_2_), 6.40 (*d*, *J* = 2.6 Hz, 1H, H3), 6.54 (*dd*, *J* = 8.9, 2.6 Hz, 1H, H5), 7.49–7.53 (*m*, 2H, 2 × H(Ar′)), 7.68 (*d*, *J* = 8.9 Hz, 1H, H6), 8.93–8.96 (*m*, 2H, 2 × H(Ar′)), and 12.70 (*s*, 1H, OH). ^13^C NMR (100 MHz, CDCl_3_): *δ* = 26.29 (COCH_3_), 70.37 (CH_2_), 101.81 (C(Ar)), 107.81 (C(Ar)), 114.67 (C(Ar)), 129.34 (2 × C(Ar′)), 129.53 (2 × C(Ar′)), 132.59 (C(Ar)), 140.73 (C(Ar)), 164.06 (C(Ar)), 165.04 (C(Ar)), 192.16 (CO), and 202.73 (COCH_3_).

2‐(4‐Acetyl‐3‐hydroxyphenoxy)‐1‐(3‐methoxyphenyl)ethan‐1‐one (**3k**): Yield: 82.4%. 2‐Bromo‐3′‐methoxyacetophenone as α‐haloketone. Purified by silica gel flash column chromatography with CH_2_Cl_2_. ^1^H NMR (400 MHz, CDCl_3_): *δ* = 2.55 (*s*, 3H, CH_3_), 3.87 (*s*, 3H, CH_3_), 5.32 (*s*, 2H, CH_2_), 6.38 (*d*, *J* = 2.5 Hz, 1H, H3), 6.54 (*dd*, *J* = 8.9, 2.6 Hz, 1H, H5), 7.16–7.19 (*m*, 1H, H(Ar′)), 7.40–7.44 (m, 1H, H(Ar′)), 7.49–7.50 (*m*, 1H, H(Ar′)), 7.52–7.55 (*m*, 1H, H(Ar′)), 7.65 (*d*, *J* = 8.9 Hz, 1H, H6), and 12.69 (*s*, 1H, OH). ^13^C NMR (100 MHz, CDCl_3_): *δ* = 26.28 (CH_3_), 55.54 (CH_3_), 70.35 (CH_2_), 101.81 (C(Ar)), 107.90 (C(Ar)), 112.35 (C(Ar)), 114.58 (C(Ar)), 120.38 (C(Ar)), 120.66 (C(Ar)), 129.98 (C(Ar)), 132.54 (C(Ar)), 135.48 (C(Ar)), 160.09 (C(Ar)), 164.29 (C(Ar)), 165.04 (C(Ar)), 192.80 (CO), and 202.71 (COCH_3_).

#### 4.1.3 General Procedure for the Synthesis of 4a–d, 4g, 4j, and 4k Derivates

In a vial, DMF (6 mL) was added to POCl_3_ (2 mmol) at −10°C, and the mixture was stirred for 10 min. **3a–d**, **3g**, **3j**, and **3k** (1 mmol) dissolved in DMF (6 mL) were added to the previous mixture at −10°C and stirred for 1 h. After that time, the reaction was kept at room temperature. Upon completion, the mixture was extracted with ethyl acetate. The combined organic layers were dried with anhydrous sodium sulfate, filtered, and the solvent evaporated. The crude product was purified by silica gel flash column chromatography with CH_2_Cl_2_/EtOAc (from 88.12% to 100% of EtOAc). The resulting fractions containing the desired compound were collected, and the solvent was evaporated to dryness under vacuum and recrystallized from CH_2_Cl_2_/n‐hexane to obtain the pure compound. The procedure was adapted from the literature [78].

7‐[2‐Oxo‐2‐(4‐methylphenyl)ethoxy]chromen‐4‐one‐3‐carbaldehyde (**4a**): Yield: 22.3%. ^1^H NMR (400 MHz, CDCl_3_): *δ* = 2.45 (*s*, 3H, CH_3_), 5.42 (*s*, 2H, CH_2_), 6.91 (*d*, *J* = 2.4 Hz, 1H, H8), 7.10 (*dd*, *J* = 8.9, 2.4 Hz, 1H, H6), 7.32–7.34 (*m*, 2H, 2 × H(Ar′)), 7.88–7.91 (*m*, 2H, 2 × H(Ar′)), 8.21 (*d*, *J* = 8.9 Hz, 1H, H5), 8.45 (*s*, 1H, H2), and 10.36 (*s*, 1H, CHO). ^13^C NMR (100 MHz, CDCl_3_): *δ* = 21.84 (CH_3_), 70.65 (CH_2_), 102.50 (C(Ar)), 115.55 (C(Ar)), 119.51 (C(Ar)), 120.30 (C(Ar)), 127.83 (C(Ar)), 128.15 (2 × C(Ar′)) 129.77 (2 × C(Ar′)), 131.53 (C(Ar)), 145.57 (C(Ar)), 157.73 (C(Ar)), 160.27 (C(Ar)), 175.20 (C(Ar)), 188.78 (CHO), and 192.24 (CO). ESI/MS *m/z* (%, fragment): 323.0912 (100).

7‐[2‐Oxo‐2‐(4‐methoxyphenyl)ethoxy]chromen‐4‐one‐3‐carbaldehyde (**4b**): Yield: 34.5%. ^1^H NMR (400 MHz, CDCl_3_): *δ* = 3.90 (*s*, 3H, OCH_3_), 5.39 (*s*, 2H, CH_2_), 6.91 (*d*, *J* = 2.4 Hz, 1H, H8), 6.97–7.01 (*m*, 2H, 2 x H(Ar′)), 7.10 (*dd*, *J* = 8.9, 2.4 Hz, 1H, H6), 7.96–8.00 (*m*, 2H, 2 × H(Ar′)), 8.20 (*d*, *J* = 8.9 Hz, 1H, H5), 8.44 (*s*, 1H, H2), and 10.36 (*s*, 1H, CHO). ^13^C NMR (100 MHz, CDCl_3_): *δ* = 55.63 (OCH_3_), 70.58 (CH_2_), 102.49 (C(Ar)), 114.29 (2 × C(Ar′)), 115.58 (C(Ar′)), 119.47 (C(Ar)), 120.28 (C(Ar)), 127.00 (C(Ar)), 127.79 (C(Ar)), 130.46 (2 x C(Ar′)), 157.73 (C(Ar)), 160.28 (C(Ar)), 163.32 (C(Ar)), 164.49 (C(Ar)), 175.19 (C(Ar)), 188.78 (CHO), and 191.14 (CO). ESI/MS *m/z* (%, fragment): 339.0860 (100).

7‐[2‐Oxo‐2‐(4‐bromophenyl)ethoxy]chromen‐4‐one‐3‐carbaldehyde (**4c**): Yield: 36.1%. ^1^H NMR (400 MHz, DMSO): *δ* = 5.81 (*s*, 2H, CH_2_), 7.24 (*dd*, *J* = 8.9, 2.4 Hz, 1H, H6), 7.39 (*d*, *J* = 2.4 Hz, 1H, H8), 7.81–7.83 (*m*, 2H, 2 × H(Ar′)), 7.96–7.98 (*m*, 2H, 2 × H(Ar′)), 8.06 (*d*, *J* = 8.9 Hz, 1H, H5), 8.85 (*s*, 1H, H2), and 10.11 (s, 1H, CHO). ^13^C NMR (100 MHz, DMSO): *δ* = 71.30 (CH_2_), 103.16 (C(Ar)), 116.32 (C(Ar)), 118.94 (C(Ar)), 120.34 (C(Ar)), 127.23 (C(Ar)), 128.53 (C(Ar)), 130.42 (2 × C(Ar′)), 132.37 (2 × C(Ar′)), 133.62 (C(Ar)), 157.79 (C(Ar)), 163.60 (C(Ar)), 163.65 (C(Ar)), 174.59 (C(Ar)), 189.01 (CHO), and 193.24 (CO). ESI/MS *m/z* (%, fragment): 386.9861 (100).

7‐[2‐Oxo‐2‐(4‐fluorophenyl)ethoxy]chromen‐4‐one‐3‐carbaldehyde (**4d**): Yield: 25.3%. ^1^H NMR (400 MHz, DMSO): *δ* = 5.82 (*s*, 2H, CH_2_), 7.24 (*dd*, *J* = 8.9, 2.4 Hz, 1H, H6), 7.38 (*d*, *J* = 2.4 Hz, 1H, H8), 7.41–7.46 (*m*, 2H, 2 × H(Ar′)), 8.06 (*d*, *J* = 8.9 Hz, 1H, H5), 8.11–8.15 (*m*, 2H, 2 × H(Ar′)), 8.85 (*s*, 1H, H2), and 10.12 (*s*, 1H, CHO). ^13^C NMR (100 MHz, DMSO): *δ* = 71.25 (CH_2_), 103.15 (C(Ar)), 116.29 (C(Ar)), 116.41 (*d*, *J*_CF_ = 18.7 Hz, C3′, C5′), 118.92 (C(Ar)), 120.34 (C(Ar)), 127.22 (C(Ar)), 131.40 (*d*, *J*_CF_ = 2.8 Hz, C1′), 131.51 (*d*, *J*_CF_ = 9.6 Hz, C2′, C6′), 157.80 (C(Ar)), 163.59 (C(Ar)), 163.70 (C(Ar)), 165.90 (*d*, *J*_CF_ = 252.6 Hz, C4′), 189.02 (CHO), and 192.56 (CO). ESI/MS *m/z* (%, fragment): 327.0661 (100).

7‐[2‐Oxo‐2‐(4‐phenylphenyl)ethoxy]chromen‐4‐one‐3‐carbaldehyde (**4g**): Yield: 17.4%. ^1^H NMR (400 MHz, CDCl_3_): *δ* = 5.47 (s, 2H, CH_2_), 6.94 (*d*, *J* = 2.4 Hz, 1H, H8), 7.13 (*dd*, *J* = 8.9, 2.4 Hz, 1H, H6), 7.42–7.46 (*m*, 1H, H(Ar)), 7.47–7.52 (*m*, 2H, 2 × H(Ar)), 7.63–7.66 (*m*, 2H, 2 × H(Ar)), 7.74–7.77 (*m*, 2H, 2 × H(Ar)), 8.06–8.09 (*m*, 2H, 2 × H(Ar)), 8.23 (*d*, *J* = 8.9 Hz, 1H, H5), 8.46 (*s*, 1H, H2), and 10.37 (*s*, 1H, CHO). ^13^C NMR (100 MHz, CDCl_3_): *δ* = 70.79 (CH_2_), 102.55 (C(Ar)), 115.54 (C(Ar)), 119.61 (C(Ar)), 120.33 (C(Ar)), 127.31 (2 × C(Ar)), 127.69 (2 × C(Ar)), 127.91 (C(Ar)), 128.68 (C(Ar)), 128.68 (2 × C(Ar)), 129.11 (2 × C(Ar)), 132.64 (C(Ar)), 139.45 (C(Ar)), 147.20 (C(Ar)), 157.75 (C(Ar)), 160.27 (C(Ar)), 163.21 (C(Ar)), 175.21 (C(Ar)), 188.77 (CHO), and 192.28 (CO). ESI/MS *m/z* (%, fragment): 385.1068 (100).

7‐[2‐Oxo‐2‐(4‐chlorophenyl)ethoxy]chromen‐4‐one‐3‐carbaldehyde (**4j**): Yield: 23.0%. ^1^H NMR (400 MHz, CDCl_3_): *δ* = 5.39 (*s*, 2H, CH_2_), 6.92 (*d*, *J* = 2.4 Hz, 1H, H8), 7.09 (*dd*, *J* = 8.9, 2.4 Hz, 1H, H6), 7.50–7.53 (*m*, 2H, 2 × H(Ar)), 7.93–7.96 (*m*, 2H, 2 × H(Ar)), 8.22 (*d*, *J* = 8.9 Hz, 1H, H5), 8.45 (*s*, 1H, H2), and 10.36 (*s*, 1H, CHO). ^13^C NMR (100 MHz, CDCl_3_): *δ* = 70.72 (CH_2_), 102.54 (C8), 115.43 (C6), 119.70 (C(Ar)), 120.34 (C(Ar)), 127.96 (C5), 129.48 (2 × C(Ar)), 129.53 (2 × C(Ar)), 132.30 (C(Ar)), 141.06 (C(Ar)), 157.71 (C(Ar)), 160.30 (C2), 162.98 (C(Ar)), 175.15 (C(Ar)), 188.71 (CHO), and 191.76 (CO). ESI/MS *m/z* (%, fragment): 343.0366 (100).

7‐[2‐Oxo‐2‐(3‐methoxyphenyl)ethoxy]chromen‐4‐one‐3‐carbaldehyde (**4k**): Yield: 26.0%. ^1^H NMR (400 MHz, CDCl_3_): *δ* = 3.88 (*s*, 3H, OCH_3_), 5.43 (*s*, 2H, CH_2_), 6.91 (*d*, *J* = 2.4 Hz, 1H, H8), 7.10 (*dd*, *J* = 8.9, 2.4 Hz, 1H, H6), 7.19–7.22 (*m*, 1H, H(Ar′)), 7.42–7.46 (*m*, 1H, H(Ar′)), 7.51–7.52 (*m*, 1H, H(Ar′)), 7.55–7.57 (*m*, 1H, H(Ar′)), 8.22 (*d*, *J* = 8.9 Hz, 1H, H5), 8.45 (*s*, 1H, H2), and 10.37 (*s*, 1H, CHO). ^13^C NMR (100 MHz, CDCl_3_): *δ* = 55.57 (OCH_3_), 70.73 (CH2), 102.52 (C(Ar)), 112.48 (C(Ar)), 115.52 (C(Ar)), 119.58 (C(Ar)), 120.32 (C(Ar)), 120.36 (C(Ar)), 120.80 (C(Ar)), 127.88 (C(Ar)), 130.10 (C(Ar)), 135.25 (C(Ar)), 157.73 (C(Ar)), 160.19 (C(Ar)), 160.28 (C(Ar)), 163.19 (C(Ar)), 175.20 (C(Ar)), 188.77 (CHO), and 192.47 (CO). ESI/MS *m/z* (%, fragment): 339.0860 (100).

### 4.2 Molecular Modeling

The modeling procedures followed the validated protocol reported by Sequeira et al. [16], which was successfully applied in similar studies.

#### 4.2.1 Ligand Preparation

Maestro GUI software [79] was used to generate theoretical 3D models of the compounds. The ligand's most stable conformation was determined by molecular mechanics conformational analysis performed with Macromodel software version 9.2 [80], considering Merck Molecular Force Fields (MMFFs) [81] as the force field and solvent effects by adopting the Generalized Born/Surface Area (GB/SA) water implicit solvation model [82]. The simulations were performed allowing 5000 steps Monte Carlo analysis with the Polak–Ribier Conjugate Gradient (PRCG) method, and a convergence criterion of 0.05 kcal/(mol Å) was used. All other parameters were left as default.

#### 4.2.2 Protein Preparation

The coordinates for *h*CA isoform enzymes were obtained from the RCSB Protein Data Bank [83] (PDB codes 5FL4 [84], for isoform IX, and 5MSA [85], for isoform XII). These 3D structures are high‐resolution x‐ray models, and the alignment with the other 3D structure did not highlight any significant difference to justify the use of an ensemble docking approach. The Maestro Protein Preparation Wizard protocol was applied to prepare the proteins. The original water molecules and ligands were removed. The Gln and Asn residues were analyzed and oriented with the best terminal amide position. Likewise, the best His tautomer was selected based on the best orientation.

#### 4.2.3 Docking Experiments

Quantum mechanics‐polarized ligand (QMPL) Docking was used for the molecular docking studies, applying the validated protocol. The validation included experiments of re‐crossdocking, which were extended to more complexes (Table S6) [58, 62, 63]. Grids on each isoform were defined around the refined structure by centering on crystallized ligands. The other settings were left as default.

#### 4.2.4 Post‐Docking Experiments

The best pose complexes were then minimized to consider the induced fit phenomena and used to analyze the ligand binding mode. 10.000 steps of the Polak‐Ribier conjugate gradient (PRCG) minimization method were conducted on the top‐ranked theoretical complexes using OPLS_2005 force field. The optimization process was performed up to the derivative convergence criterion equal to 0.1 kcal/mol [86].

### 4.3 Drug‐Like Properties

#### 4.3.1 Theoretical Prediction

Drug‐like properties of compounds were theoretically predicted using QikProp software and reported in Tables S1–S4.

#### 4.3.2 Evaluation of Drug‐Like Properties by Biomimetic HPLC

All chromatographic experiments were carried out on a NEXERA‐i LC‐2040C ultra‐high‐performance liquid chromatography (UHPLC) (Shimadzu, Kyoto, Japan) equipped with a diode‐array detector and controlled by the LabSolution system (version 5.90 Shimadzu).

#### 4.3.3 Prediction of Lipophilicity

##### 4.3.3.1 Chromatographic Hydrophobicity Index (CHI) Determination of the Compounds Under Study

The values at pH 7.4 were determined using an experimental protocol already described by our group [87]. The CHI values were assessed from experimental retention times (*t*_R_) of the samples and by correlation with the data of a mixture of reference compounds using a Luna C18 (2) column (150 × 4.6 mm, 5 µm, Phenomenex, CA, USA). Stock solutions of compounds in DMSO (10 mM) were diluted in acetonitrile:water (1:1) to obtain a final concentration of 250 μM. The mobile phase A was 30 mM ammonium acetate aqueous solution (pH 7.4), and mobile phase B was acetonitrile. The following gradient program was applied: 1−7 min 0%–100% B, 7−10 min 100% B, and 10–12 min 100%–0% B. The flow rate was 1 mL/min, and the injection volume was 20 μL. The system was calibrated using known standards with reported values of CHI (Figure S1 and Table S7) [87]. The values of CHI obtained for each sample were then converted to LogP as described by Valko et al. [70].

#### 4.3.4 Prediction of Lipophilicity of Membrane Binding Using Immobilized Artificial Membrane (IAM)

For the measurements of the compounds under study, interactions with phospholipids, the *t*_R_ of the samples has been measured using IAM.PC.DD2 100 × 4.6 mm column with 10 µM diameter and 300 Å pore size particles. The mobile phases were the same as those described for the CHI determination. The following gradient program was applied: 1−7 min 0%–80% B, 7−9 min 80% B, and 9−10 min 80%–0% B. The flow rate was 1 mL/min, and the injection volume was 20 μL. The system was calibrated using known standards with reported values of CHI(IAM) (Figure S2 and Table S8) [69]. The values of CHI(IAM) obtained for each sample were then converted to logKpcell values as described by Valko et al. [70].

#### 4.3.5 Prediction of the Interaction With Human Serum Albumin

The interaction of the compounds under study with human serum albumin [72] has been measured using a commercially available chemically bonded HSA (Chiralpak‐HSA) HPLC column with the dimensions of 50 × 3 mm with 5 µM particle size obtained from HiChrom Ltd, Reading, UK. The mobile phase was 30 mM ammonium acetate with the pH adjusted to 7.4 (Phase A) and isopropanol (Phase B). The following gradient program was applied: 0−4 min 0%–30% B, 4–14 min 30% B, and 14−15 min 30%–0% B. The flow rate was 1 mL/min, and the injection volume was 20 μL. The system was calibrated using known standards with reported values of percentage of binding with HAS (%HSA) (Figure S3 and Table S9) [69]. The values of %HSA as well as the values of unbound volume distribution (Vdu) and maximum drug efficiency (DEmax) were calculated as described in the literature [70].

### 4.4 Biological Assays

#### 4.4.1 CA Inhibition Assay

The CA catalyzed CO_2_ hydration/inhibition was measured by using a stopped‐flow instrument as previously described [88]. Initial rates of the CA‐catalyzed CO_2_ hydration reaction were followed for 10–100 s. The CO_2_ concentrations ranged from 1.7 to 17 mM for the determination of the inhibition constants. For each inhibitor, at least six traces of the initial 5%–10% of the reaction were used for assessing the initial velocity. The uncatalyzed rates were subtracted from the total observed rates. Stock solutions of inhibitors (10 mM) and dilutions up to 0.01 nM were prepared in distilled‐deionized water. Inhibitor and enzyme solutions were preincubated together for 15 min at room temperature before assay, to allow for the formation of the E–I complex. The inhibition constants were obtained by nonlinear least‐squares methods using PRISM 3 as reported earlier and represent the mean from at least three different determinations. *h*CA I, *h*CA II, *h*CA IX, and *h*CA XII (catalytic domain) were recombinant proteins produced in‐house using our standardized protocol, and their concentration in the assay system was in the range of 3–10 nM. AAZ was used as a reference CA inhibitor [89, 90, 91].

### 4.5 Cytotoxicity Profile

#### 4.5.1 Chemicals and Reagents

All reagents used were of analytical grade or of the highest grade available. Sulforhodamine B (SRB), trypan blue solution [0.4% (w/v)], and minimum essential medium (MEM, M0643) with 1 g/L glucose were obtained from Sigma‐Aldrich (St. Louis, MO, USA). Reagents used in cell culture, including heat‐inactivated fetal bovine serum (FBS), 0.05 or 0.25% trypsin/1 mM EDTA, antibiotic (10,000 U/mL penicillin, 10,000 μg/mL streptomycin), and phosphate‐buffered saline solution (PBS) were purchased from PanBiotech (Aidenbach, Germany). Resazurin sodium salt was acquired from TCI (Zwijndrecht, Belgium). Dimethylsulfoxide (DMSO), absolute ethanol, and acetic acid were obtained from Merck (Darmstadt, Germany).

#### 4.5.2 Cell Culture Conditions

##### 4.5.2.1 HepG2 Cells

Human HepG2 cells (ATCC, ATCC‐HB‐8065, Lot: 70047955) were routinely cultured in 25‐cm^2^ flasks using MEM (M0625) with 1 mM glucose, supplemented with 1.5 g/L of sodium bicarbonate, 0.11 g/L of sodium pyruvate, 10% FBS, and 100 U/mL penicillin and 100 μg/mL streptomycin. Cells were maintained at 37°C in a humidified incubator with 5% CO_2_ and passaged weekly by trypsinization (0.25% trypsin/1 mM EDTA). In all experiments, the cells were seeded at a density of 60,000 cells/cm^2^ in 96‐well plates and grown for 24 h before treatments. The cells used in all the experiments were between the 12nd and 20th passages.

##### 4.5.2.2 Caco‐2 Cells

Caco‐2 cells (ATCC‐HTB‐37, Lot: 70046148) were routinely cultured in 75‐cm^2^ flasks using DMEM with 4.5 g/L glucose, supplemented with 10% heat inactivated FBS, 100 μM NEAA, 100 U/mL penicillin, and 100 μg/mL streptomycin. The cells were maintained in a 5% CO_2_–95% air atmosphere, at 37°C, and the medium was changed every 2 days. Cultures were passed weekly by trypsinization with 0.25% trypsin/1 mM EDTA. The cells used in all the experiments were taken between the second and ninth passages. In all experiments, cells were seeded onto 96‐well plates (60,000 cells/cm^2^) and used 3 days after seeding, when confluence was reached.

#### 4.5.3 Cell Viability Evaluation

##### 4.5.3.1 Incubation With Test Compounds

Both types of cells were exposed to a range of concentrations from the test compounds (1–20 μM) for 24 h, with the cellular viability evaluated by measurement of metabolic activity (resazurin reduction) and cell mass (SRB) assays [92], and the data were compared with results of untreated cells (% of control). Stock solutions of the test compounds (10 mM) were prepared in DMSO, and each compound solution was diluted in cell medium to reach the desired concentration. The concentration of DMSO per well was always lower than 0.2%. All studies were performed in triplicate from at least four independent experiments, and the data are presented as means ± standard deviation (SD) of percentage of control data (control = 100%).

##### 4.5.3.2 Cell Metabolic Activity Evaluation

After incubation time, the cell culture medium was removed and replaced with a fresh medium containing resazurin (10 μg/mL). The assay conditions have been previously reported [93].

##### 4.5.3.3 Cell Mass Evaluation

After incubation, the cell culture medium was removed, the wells were rinsed with PBS (1×), and cells were fixed by adding 1% acetic acid in 100% methanol for at least 2 h at −20°C. The assay conditions have been previously described [93].

## Conflicts of Interest

The authors declare no conflicts of interest.

## Supporting information

InChI Sequeira et al ArchParmazie.

Supporting Sequeira et al ArchPharm Revised.

## Data Availability

The data that support the findings of this study are available from the corresponding author upon reasonable request.
