# Supplementary material for: Chromones as Nonclassical Inhibitors of Carbonic Anhydrase IX and XII Isoforms: Probing Chromone‐Based Derivatives
Source: Arch Pharm (Weinheim). 2026 Mar 16;359(3):e70224. doi: 10.1002/ardp.70224 (PMC12991041; doi:10.1002/ardp.70224)
Supplement: Supplementary file 1 — InChI Sequeira et al ArchParmazie. [file ARDP-359-e70224-s001.doc]

**Supplemental Material: Novel Compounds and Biological Screening Results**

Title of Manuscript:

Chromones as non-classical inhibitors of Carbonic anhydrase IX and XII isoforms: probing chromone-based derivatives

Authors:

Lisa Sequeira^1^, Simona Distinto^2^, Carlos Fernandes^1,3^, Erica Sanna^2^, Rita Meleddu^2^, Marco Gaspari^4^, Filippo Cottiglia^2^, Alessia Onali^2^, Andrea Angeli^5^, Fernanda Borges^1,3^, Eugenio Uriarte^6^, Stefano Alcaro^7^, Claudiu T. Supuran^5^*, and Elias Maccioni^2^*

Affiliations:

1 MedInUP, Department of Biomedicine, Faculty of Medicine, University of Porto, Porto, Portugal

2 Department of Life and Environmental Sciences, University of Cagliari, Monserrato, Italy

3 Department of Chemistry and Biochemistry, Faculty of Sciences, University of Porto, Porto, Portugal

4 Research Centre for Advanced Biochemistry and Molecular Biology, Department of Experimental and Clinical Medicine, “Magna Græcia” University of Catanzaro, Catanzaro, Italy

5 Department NEUROFARBA, Section of Pharmaceutical Sciences, University of Florence, Sesto Fiorentino, Florence, Italy

6 Department of Organic Chemistry, Faculty of Pharmacy, University of Santiago de Compostela, Santiago de Compostela, Spain

7 Department of Health Sciences, “Magna Græcia” University of Catanzaro, Catanzaro, Italy

Corresponding author – full address:

Prof, Elias Maccioni, Department of Life and Environmental Sciences, Section of Drug Sciences, University of Cagliari, A Block, Cittadella Universitaria, 09042 Monserrato, Italy-Email: elias.maccioni@unica.it

Prof, Claudiu T. Supuran, Department NEUROFARBA, Section of Pharmaceutical Sciences, University of Florence, via U. Schiff 6, 50019 Sesto Fiorentino, Florence, Italy-Email: claudiu.supuran@unifi.it

| **Compound No.** | **InChI** | **Biological Activity (*K*_i_ μM)^a^** | | | |
| --- | --- | --- | --- | --- | --- |
|  |  | hCA I | hCA II | hCA IX | hCA XII |
| **4a** | InChI=1S/C19H14O5/c1-12-2-4-13(5-3-12)17(21)11-23-15-6-7-16-18(8-15)24-10-14(9-20)19(16)22/h2-10H,11H2,1H3 | > 100 | > 100 | 0.44 | 0.33 |
| **4b** | InChI=1S/C19H14O6/c1-23-14-4-2-12(3-5-14)17(21)11-24-15-6-7-16-18(8-15)25-10-13(9-20)19(16)22/h2-10H,11H2,1H3 | > 100 | > 100 | > 100 | > 100 |
| **4c** | InChI=1S/C18H11BrO5/c19-13-3-1-11(2-4-13)16(21)10-23-14-5-6-15-17(7-14)24-9-12(8-20)18(15)22/h1-9H,10H2 | > 100 | > 100 | > 100 | > 100 |
| **4d** | InChI=1S/C18H11FO5/c19-13-3-1-11(2-4-13)16(21)10-23-14-5-6-15-17(7-14)24-9-12(8-20)18(15)22/h1-9H,10H2 | > 100 | > 100 | > 100 | > 100 |
| **4g** | InChI=1S/C24H16O5/c25-13-19-14-29-23-12-20(10-11-21(23)24(19)27)28-15-22(26)18-8-6-17(7-9-18)16-4-2-1-3-5-16/h1-14H,15H2 | > 100 | > 100 | 0.86 | 0.69 |
| **4j** | InChI=1S/C18H11ClO5/c19-13-3-1-11(2-4-13)16(21)10-23-14-5-6-15-17(7-14)24-9-12(8-20)18(15)22/h1-9H,10H2 | > 100 | > 100 | 0.42 | 0.28 |
| **4k** | InChI=1S/C19H14O6/c1-23-14-4-2-3-12(7-14)17(21)11-24-15-5-6-16-18(8-15)25-10-13(9-20)19(16)22/h2-10H,11H2,1H3 | > 100 | > 100 | 0.31 | 0.24 |

^a^ The CA catalyzed CO_2_ hydration/inhibition was measured by using a stopped-flow instrument as the method previously described [1]. Initial rates of the CA-catalyzed CO_2_ hydration reaction were followed for 10 – 100 s. The CO_2_ concentrations ranged from 1.7 to 17 mM for the determination of the inhibition constants. For each inhibitor, at least six traces of the initial 5 – 10 % of the reaction were used for assessing the initial velocity. The uncatalyzed rates were subtracted from the total observed rates. Stock solutions of inhibitors (10 mM) and dilutions up to 0.01 nM were prepared in distilled-deionized water. Inhibitor and enzyme solutions were preincubated together for 15 min at room temperature before assay, to allow for the formation of the E – I complex. The inhibition constants were obtained by non-linear least-squares methods using PRISM 3 as reported earlier and represent the mean from at least three different determinations. hCA I, hCA II, hCA IX, and hCA XII (catalytic domain) were recombinant proteins produced in–house using our standardized protocol and their concentration in the assay system was in the range of 3 – 10 nM. AAZ was used as reference CA inhibitor [2-4].

Ref

1. Khalifah, R. G. "The Carbon Dioxide Hydration Activity of Carbonic Anhydrase: I. Stop-Flow Kinetic Studies On The Native Human Isoenzymes B And C".Journal of Biological Chemistry (8) 246 (1971): 2561-2573.

2. Berrino, E.; et al. "Azidothymidine “Clicked” into 1,2,3-Triazoles: First Report on Carbonic Anhydrase–Telomerase Dual-Hybrid Inhibitors".Journal of Medicinal Chemistry (13) 63 (2020): 7392-7409.

3. Pacchiano, F.; et al. "Ureido-Substituted Benzenesulfonamides Potently Inhibit Carbonic Anhydrase IX and Show Antimetastatic Activity in a Model of Breast Cancer Metastasis".Journal of Medicinal Chemistry (6) 54 (2011): 1896-1902.

4. Bilginer, S.; et al. "Novel sulphonamides incorporating triazene moieties show powerful carbonic anhydrase I and II inhibitory properties".Journal of enzyme inhibition and medicinal chemistry (1) 35 (2020): 325-329.
