# Supplementary material for: Chromones as Nonclassical Inhibitors of Carbonic Anhydrase IX and XII Isoforms: Probing Chromone‐Based Derivatives
Source: Arch Pharm (Weinheim). 2026 Mar 16;359(3):e70224. doi: 10.1002/ardp.70224 (PMC12991041; doi:10.1002/ardp.70224)
Supplement: Supplementary file 2 — Supporting Sequeira et al ArchPharm Revised. [file ARDP-359-e70224-s002.docx]

Supporting Information

Chromones as non-classical inhibitors of Carbonic anhydrase IX and XII isoforms: probing chromone-based derivatives

Lisa Sequeira, Simona Distinto, Carlos Fernandes, Erica Sanna, Rita Meleddu, Marco Gaspari, Filippo Cottiglia, Alessia Onali, Andrea Angeli, Fernanda Borges, Eugenio Uriarte, Stefano Alcaro, Claudiu T. Supuran, and Elias Maccioni

[Table S1. QikProp properties and descriptors with description and range or recommended values, from the QikPro user manual. 3](#_Toc213927149)

[Table S2. Predicted properties for the series 4. 4](#_Toc213927150)

[Table S3. Ranges of pharmacokinetics properties calculated considering 95% of drugs. 4](#_Toc213927151)

[Table S4. Predicted pharmacokinetics properties for the series 4. 4](#_Toc213927152)

[Blank ESI/MS spectra 5](#_Toc213927153)

[^1^H, ^13^C and DEPT NMR spectra of 3a 6](#_Toc213927154)

[^1^H, ^13^C and DEPT NMR spectra of 3b 7](#_Toc213927155)

[^1^H, ^13^C and DEPT NMR spectra of 3c 8](#_Toc213927156)

[^1^H, ^13^C and DEPT NMR spectra of 3d 9](#_Toc213927157)

[^1^H, ^13^C and DEPT NMR spectra of 3g 10](#_Toc213927158)

[^1^H, ^13^C and DEPT NMR spectra of 3j 11](#_Toc213927160)

[^1^H, ^13^C and DEPT NMR spectra of 3k 12](#_Toc213927161)

[^1^H, ^13^C and DEPT NMR, and ESI/MS spectra, and chromatogram of 4a 13](#_Toc213927162)

[^1^H, ^13^C and DEPT NMR, and ESI/MS spectra, and chromatogram of 4b 15](#_Toc213927163)

[^1^H, ^13^C and DEPT NMR, and ESI/MS spectra, and chromatogram of 4c 17](#_Toc213927164)

[^1^H, ^13^C and DEPT NMR, and ESI/MS spectra, and chromatogram of 4d 19](#_Toc213927165)

[^1^H, ^13^C and DEPT NMR, and ESI/MS spectra, and chromatogram of 4g 21](#_Toc213927166)

[^1^H, ^13^C and DEPT NMR, and ESI/MS spectra of, and chromatogram 4j 23](#_Toc213927167)

[^1^H, ^13^C and DEPT NMR, and ESI/MS spectra, and chromatogram of 4k 25](#_Toc213927168)

[Table S5. Calculated and experimental mass values for 4 series. 26](#_Toc213927169)

[Table S6. Cross-docking results considering the co-crystallized ligands reported in pdb complexes. Receptor coordinates were obtained considering *h*CA IX pdb code 5FL4, and *h*CA XII pdb code 5MSA. 27](#_Toc213927170)

[Figure S1. Calibration curve for CHI measurements using a mixture solution with 11 compounds with known CHI values. 28](#_Toc213927171)

[Table S7. Values obtained for the retention time of each compound present in the mixture solution for the measurements of CHI using a UHPLC system. 28](#_Toc213927172)

[Figure S2. Calibration curve for CHI(IAM) measurements using a mixture solution with 9 compounds with known CHI(IAM) values. 29](#_Toc213927173)

[Table S8. Values obtained for the retention time of each compound present in the mixture solution for the measurements of CHI(IAM) using a UHPLC system. 29](#_Toc213927174)

[Figure S3. Calibration curve of Log K calculated by Log[HSA%/(101-HAS%)] and the logarithmic of retention time of a mixture of compounds with known percentage of binding with HSA. 30](#_Toc213927175)

[Table S9. Values of retention time obtained for the compounds used in the mixture solution and the values of Log K and Log (tr) calculated. 30](#_Toc213927176)

**Physical-Chemical Property Prediction of the Best Compounds**

To estimate the drug-likeness of the compounds, we carried out in silico ADMET prediction. In particular, a selection of the predicted properties has been predicted using the QikProp software.

For each series of compounds two tables are reported. One containing the structural properties under Lipinski's rule of five (RO5) and other physicochemical properties (Table S2), and another with other properties that could affect the pharmacokinetics and the toxicity of the compounds (Table S4).

# Table S1. QikProp properties and descriptors with description and range or recommended values, from the QikPro user manual.

| **Property or Descriptor** | **Description** | **Range or Recommended Values** |
| --- | --- | --- |
| Stars | Number of property or descriptor values that fall outside the 95% range of similar values for known drugs. A large number of stars suggests that a molecule is less drug-like than molecules with few stars. The following properties and descriptors are included in the determination of stars: MW, dipole, IP, EA, SASA, FOSA, FISA, PISA, WPSA, PSA, volume, rotor, donorHB, accptHB, glob, QPpolrz, QPlogPC16, QPlogPoct, QPlogPw, QPlogPo/w, logS, QPLogKhsa, QPlogBB, metabol. | 0 – 5 |
| Rotor | Number of non-trivial (not CX3), non-hindered (not alkene, amide, small ring) rotatable bonds. | 0 – 15 |
| CNS | Predicted central nervous system activity on a –2 (inactive) to +2 (active) scale. | -2 – +2 |
| mol_MW | Molecular weight of the molecule. | 130.0 – 725.0 |
| donorHB | Estimated number of hydrogen bonds that would be donated by the solute to water molecules in an aqueous solution. Values are averages taken over a number of configurations, so they can be non-integer. | 0.0 – 6.0 |
| accptHB | Estimated number of hydrogen bonds that would be accepted by the solute from water molecules in an aqueous solution. Values are averages taken over a number of configurations, so they can be non-integer. | 2.0 – 20.0 |
| QPlogPo/w | Predicted octanol/water partition coefficient. | -2.0 – +6.5 |
| QPlogS | Predicted aqueous solubility, log S. S in mol dm^–3^ is the concentration of the solute in a saturated solution that is in equilibrium with the crystalline solid. | -6.5 – +0.5 |
| QPlogHERG | Predicted IC_50_ value for blockage of HERG K^+^ channels. | Concern below -5 |
| QPPCaco | Predicted apparent Caco-2 (colorectal adenocarcinoma) cell permeability in nm/sec. Caco-2 cells are a model for the gut-blood barrier. QikProp predictions are for non-active transport. | < 25: poor  > 500: great |
| QPlogBB | Predicted brain/blood partition coefficient. Note: QikProp predictions are for orally delivered drugs so, for example, dopamine and serotonin are CNS negative because they are too polar to cross the blood-brain barrier | -3 – +1.2 |
| QPPMDCK | Predicted apparent MDCK (Madin-Darby Canine Kidney) cell permeability in nm/sec. MDCK cells are considered to be a good mimic for the blood-brain barrier. QikProp predictions are for non-active transport. | < 25: poor  > 500: great |
| QPlogKhsa | Prediction of binding to human serum albumin. | -1.5 – +1.5 |
| PercentHuman-  OralAbsorption | Predicted human oral absorption on 0 to 100% scale. The prediction is based on a quantitative multiple linear regression model. This property usually correlates well with HumanOral-Absorption, as both measure the same property. | > 80%: high  < 25%: poor |
| SAFluorine | Solvent-accessible surface area of fluorine atoms. | 0.0 – 100.0 |
| PSA | Van der Waals surface area of polar nitrogen and oxygen atoms. | 7.0 – 200.0 |

# Table S2. Predicted properties for the series 4.

| **Compound** | **#stars** | **mol_MW** | **donorHB** | **accptHB** | **QPlogPo/w** | **#rotor** | **PSA** | **QPlogS** |
| --- | --- | --- | --- | --- | --- | --- | --- | --- |
|  | **0 – 5** | **130 – 725** | **0 – 6** | **2 – 20** | **-2 – 6.5** | **0 – 15** | **7 – 200.0** | **-6.5 – 0.5** |
| **4a** | 0 | 322.32 | 0 | 7 | 4.0 | 5 | 106.9 | -3.5 |
| **4b** | 0 | 338.32 | 0 | 8 | 3.8 | 6 | 115.2 | -3.5 |
| **4c** | 0 | 387.19 | 0 | 7 | 4.3 | 5 | 106.9 | -4.9 |
| **4d** | 0 | 326.28 | 0 | 7 | 4.0 | 5 | 106.9 | -3.6 |
| **4g** | 1 | 384.39 | 0 | 7 | 5.4 | 6 | 106.9 | -5.2 |
| **4j** | 0 | 342.74 | 0 | 7 | 4.1 | 5 | 106.9 | -4.0 |
| **4k** | 0 | 338.32 | 0 | 8 | 3.7 | 6 | 115.2 | -3.5 |

# Table S3. Ranges of pharmacokinetics properties calculated considering 95% of drugs.

| **Property** | **Recommended range** |
| --- | --- |
| **QPPCaco** | < 25 poor  >500 great |
| **QPPMDCK** | < 25 poor  >500 great |
| **PercentHumanOralAbsorption (%OA)** | > 80 % is high  < 25 % is poor |
| **QPlogHERG** | >-5 |
| **CNS** | - 2 (inactive) to +2 (active) |
| **QPlogBB** | -3.0 – 1.2 |
| **QPlogKhsa** | -1.5 – 1.5 |

# Table S4. Predicted pharmacokinetics properties for the series 4.

| **Compound** | **QPPCaco** | **QPPMDCK** | **%OA** | **KPlogHERG** | **CNS** | **QPlogBB** | **QPlogKhsa** |
| --- | --- | --- | --- | --- | --- | --- | --- |
| **4a** | 238.8 | 105.2 | 78.9 | -5.8 | -2 | -1.4 | -0.6 |
| **4b** | 238.7 | 105.2 | 77.3 | -5.8 | -2 | -1.5 | -0.8 |
| **4c** | 238.9 | 278.2 | 81.2 | -6.2 | -2 | -1.3 | -0.5 |
| **4d** | 239.0 | 189.0 | 78.4 | -5.8 | -2 | -1.3 | -0.7 |
| **4g** | 238.7 | 105.2 | 86.8 | -7.1 | -2 | -1.6 | -0.1 |
| **4j** | 238.9 | 259.2 | 79.9 | -5.8 | -2 | -1.2 | -0.6 |
| **4k** | 239.2 | 105.4 | 77.3 | -5.8 | -2 | -1.5 | -0.8 |

# Blank ESI/MS spectra

# ^1^H, ^13^C and DEPT NMR spectra of 3a


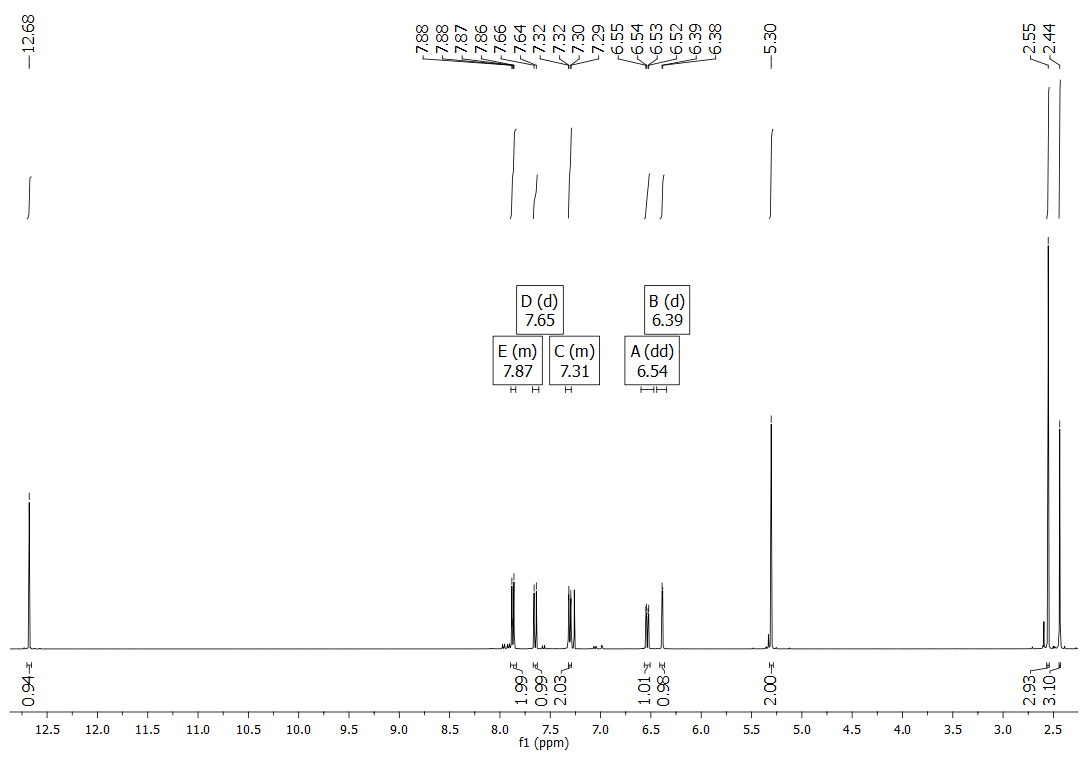


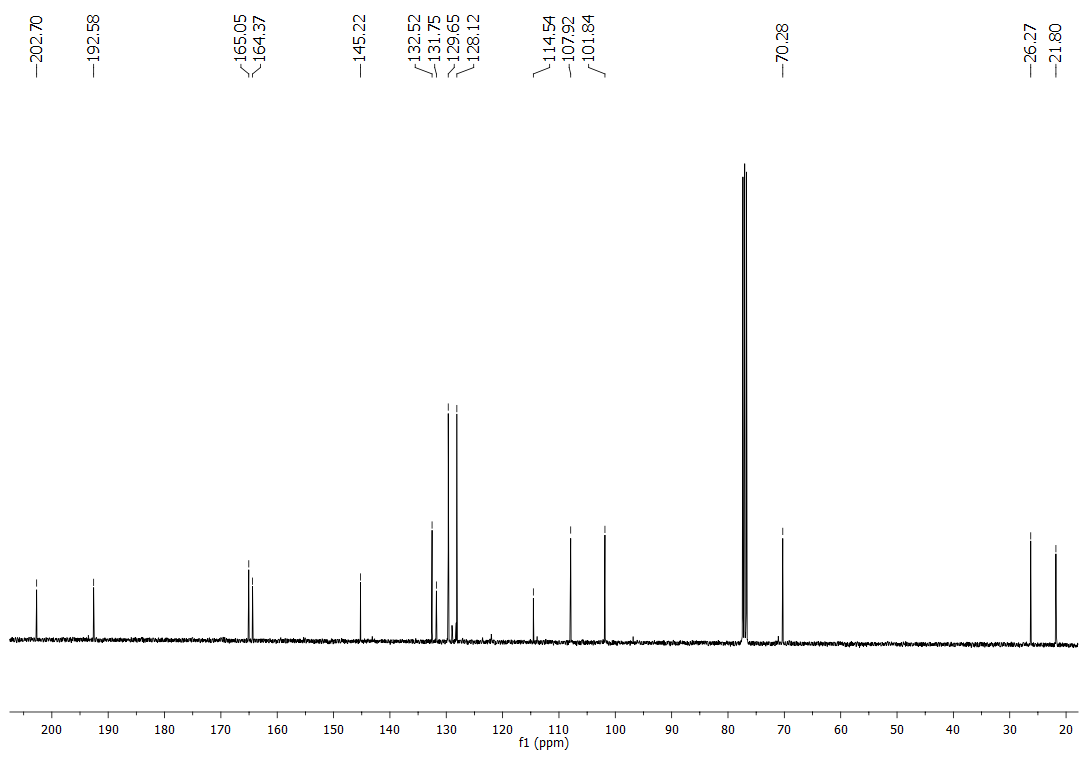


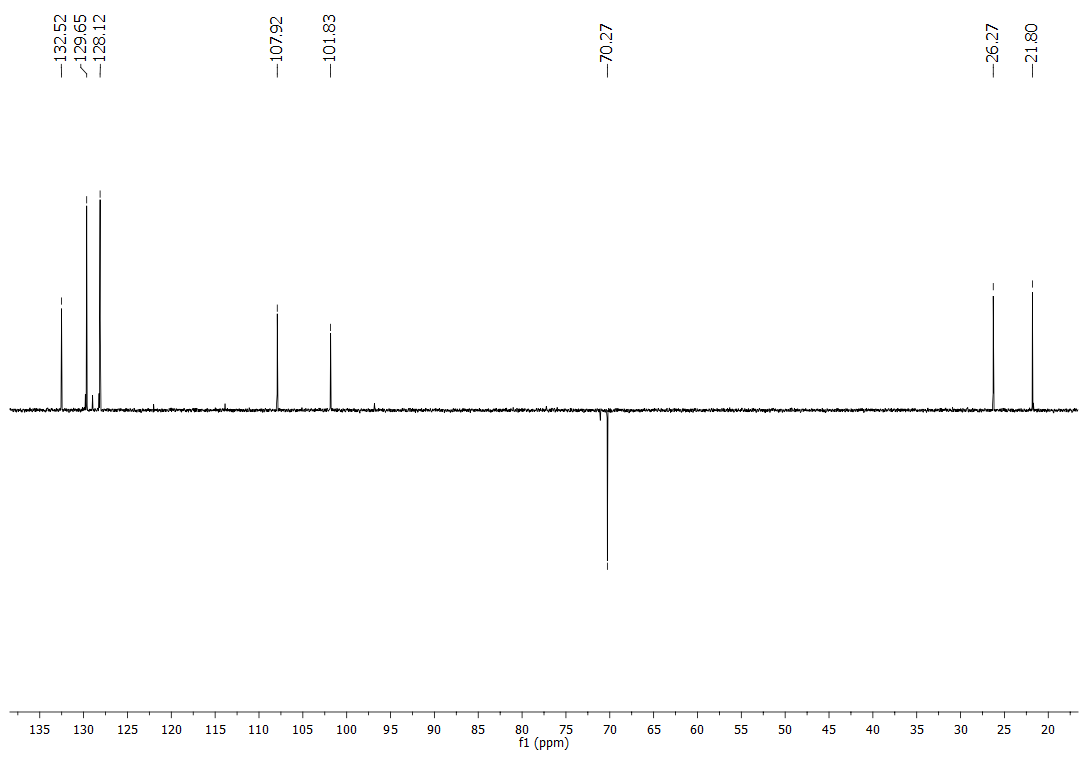


# ^1^H, ^13^C and DEPT NMR spectra of 3b


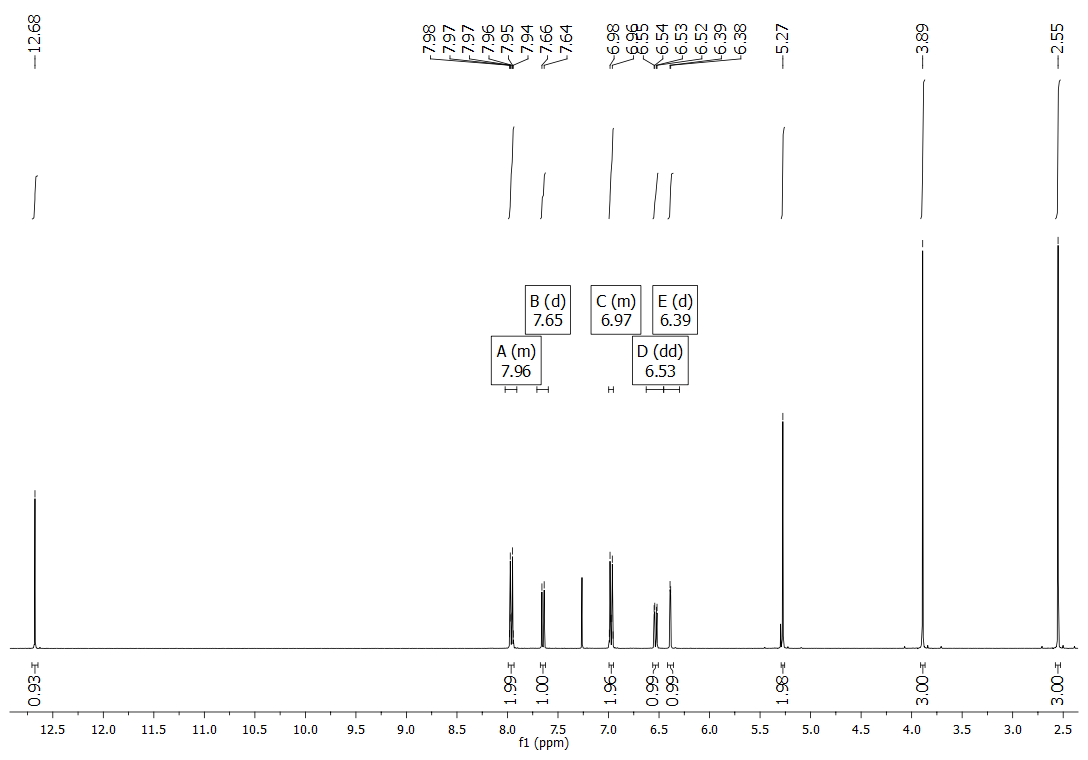


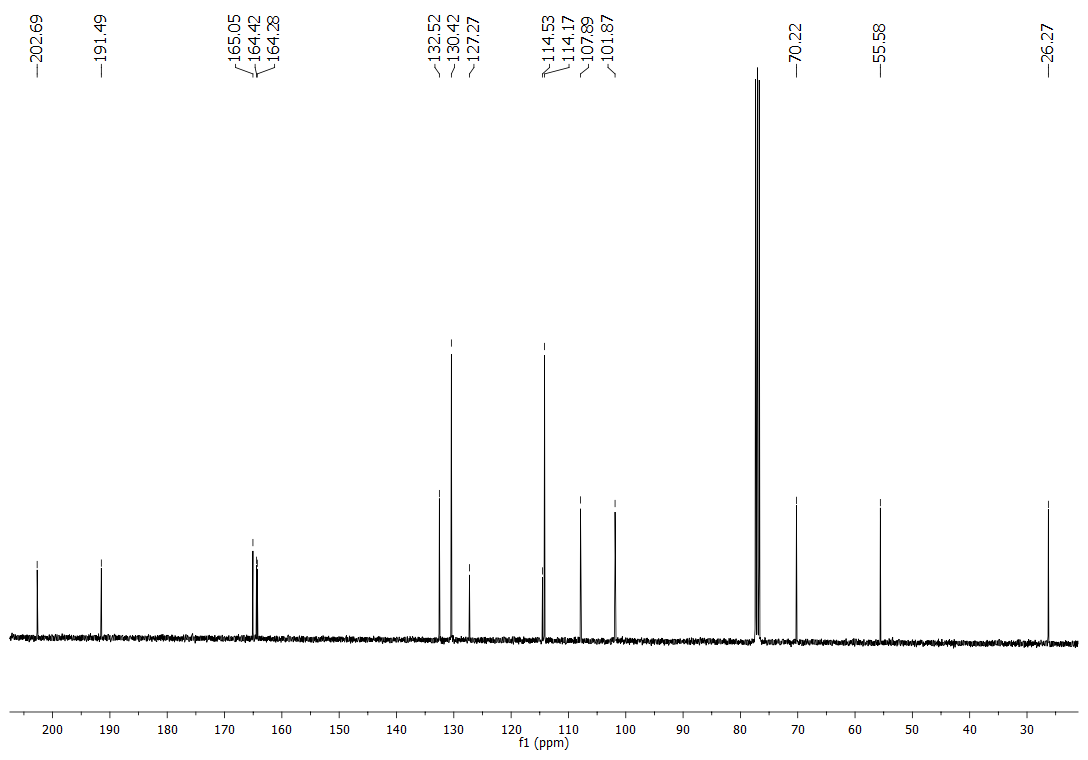


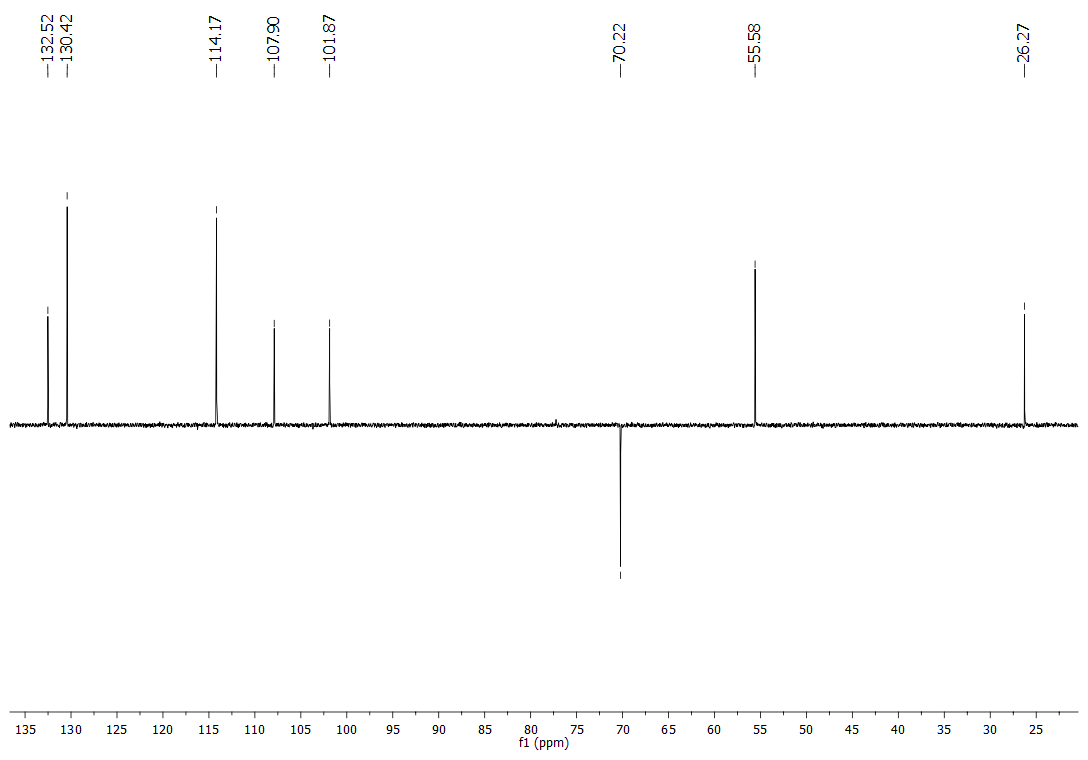


# ^1^H, ^13^C and DEPT NMR spectra of 3c


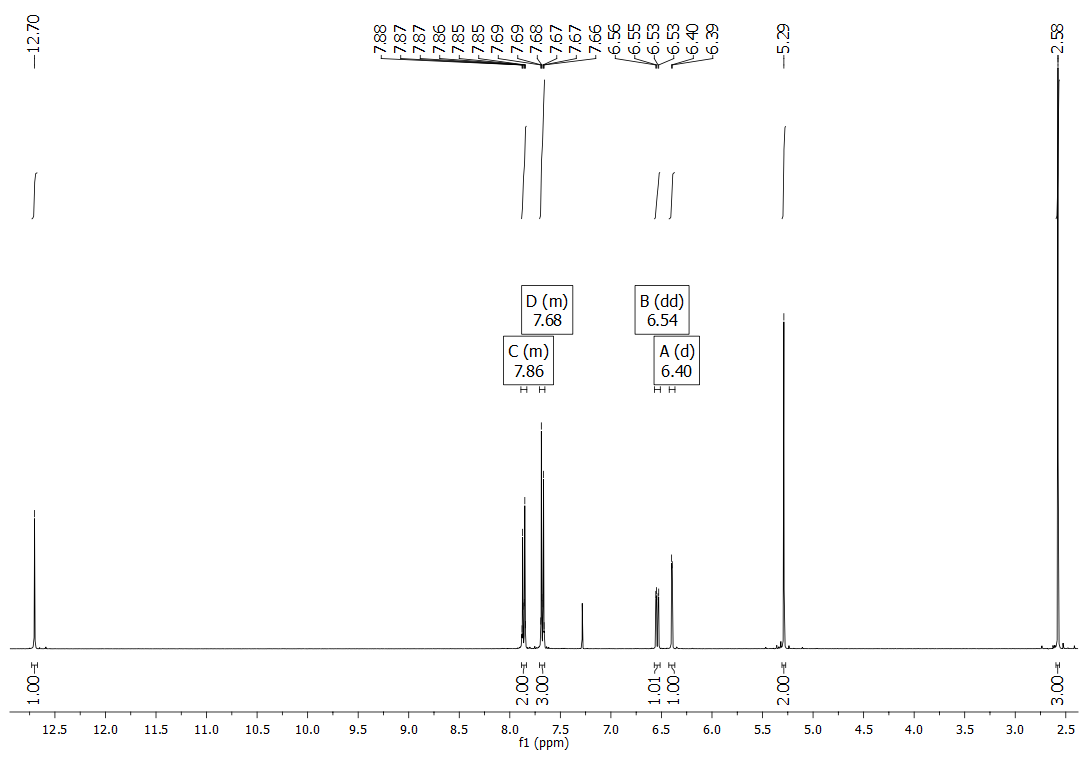


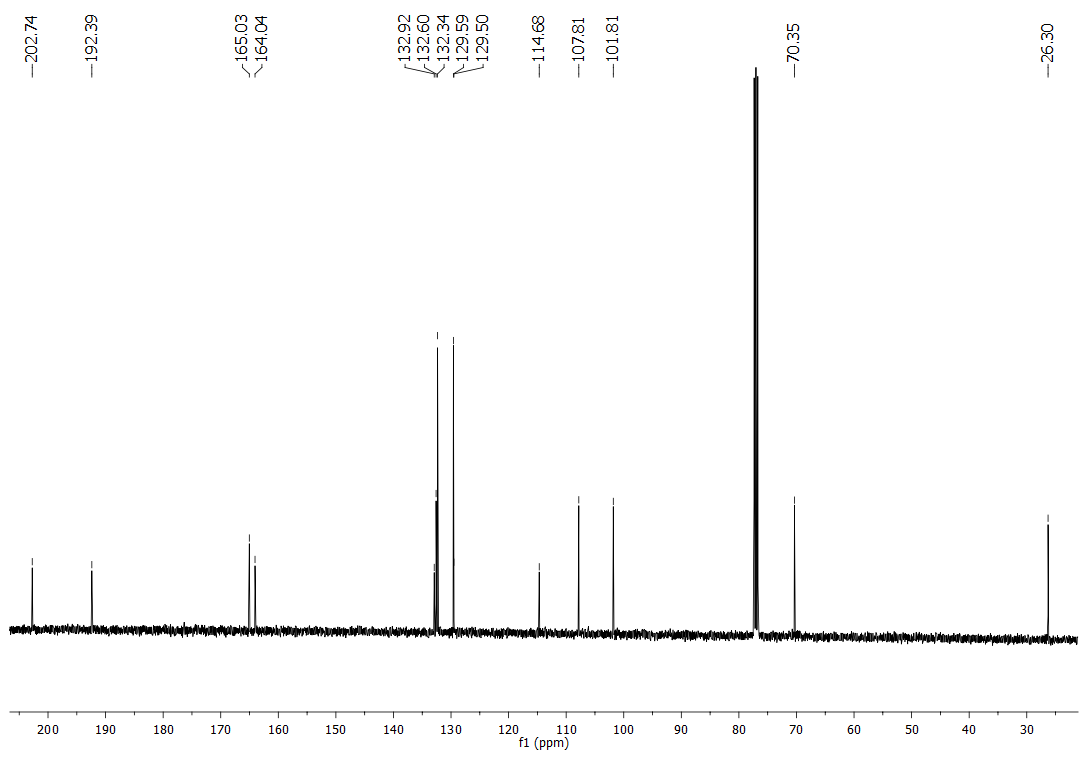


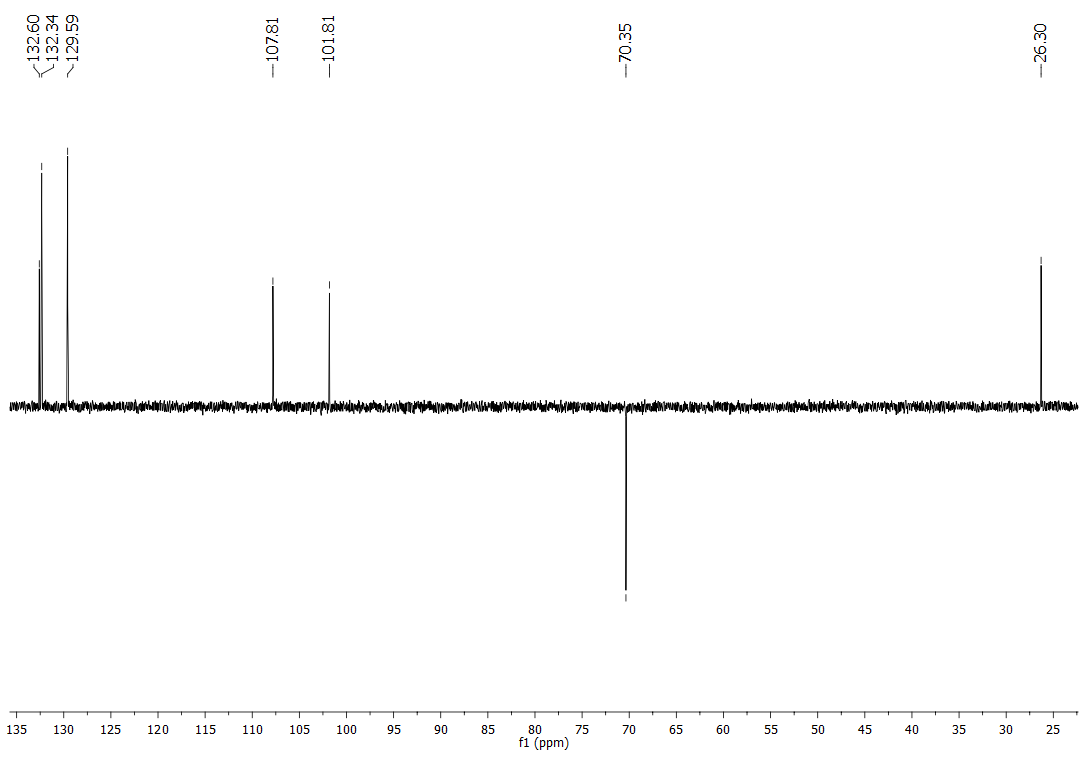


# ^1^H, ^13^C and DEPT NMR spectra of 3d


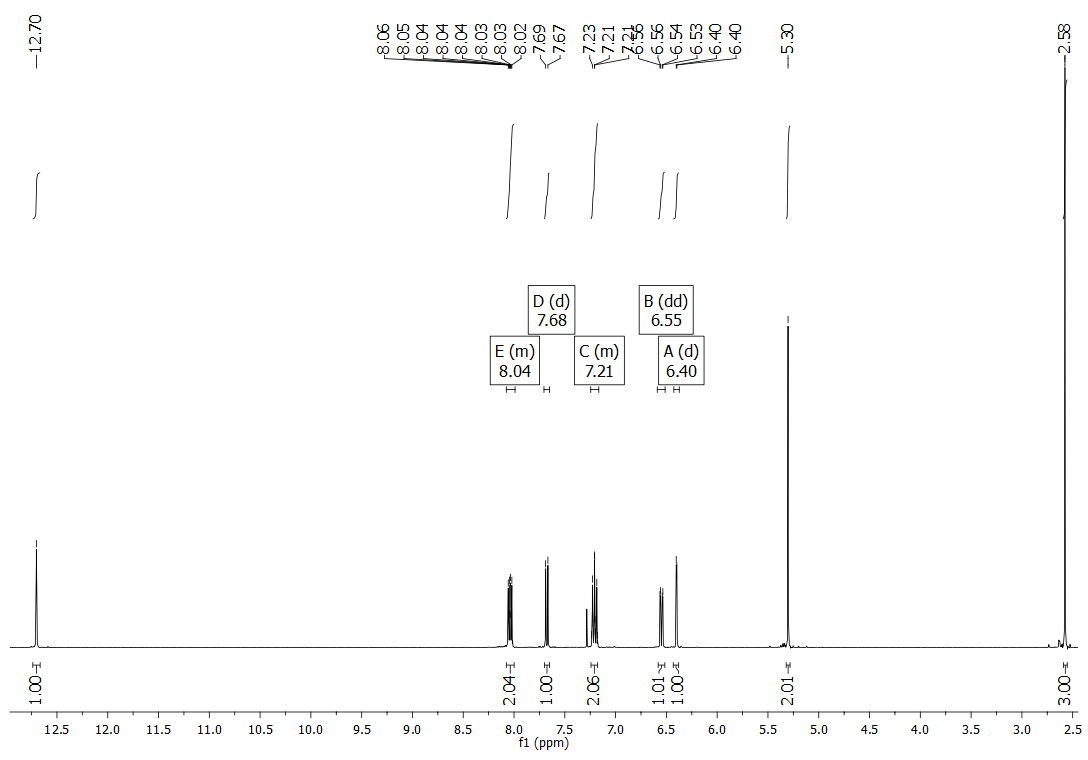


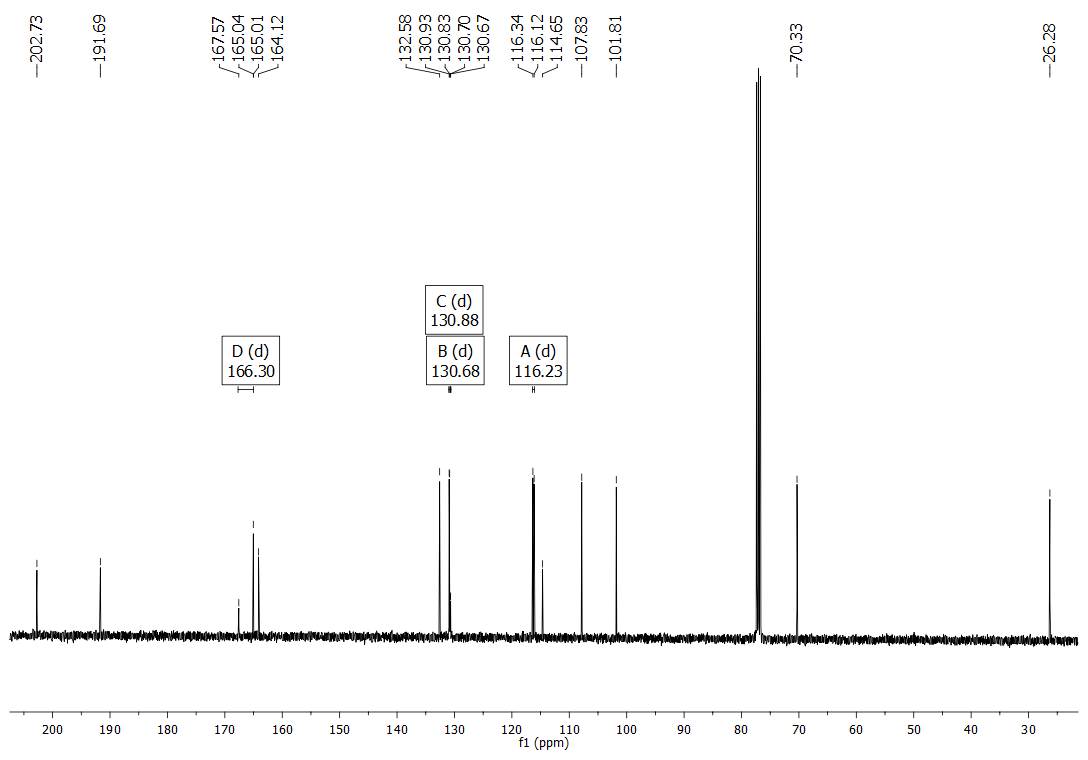


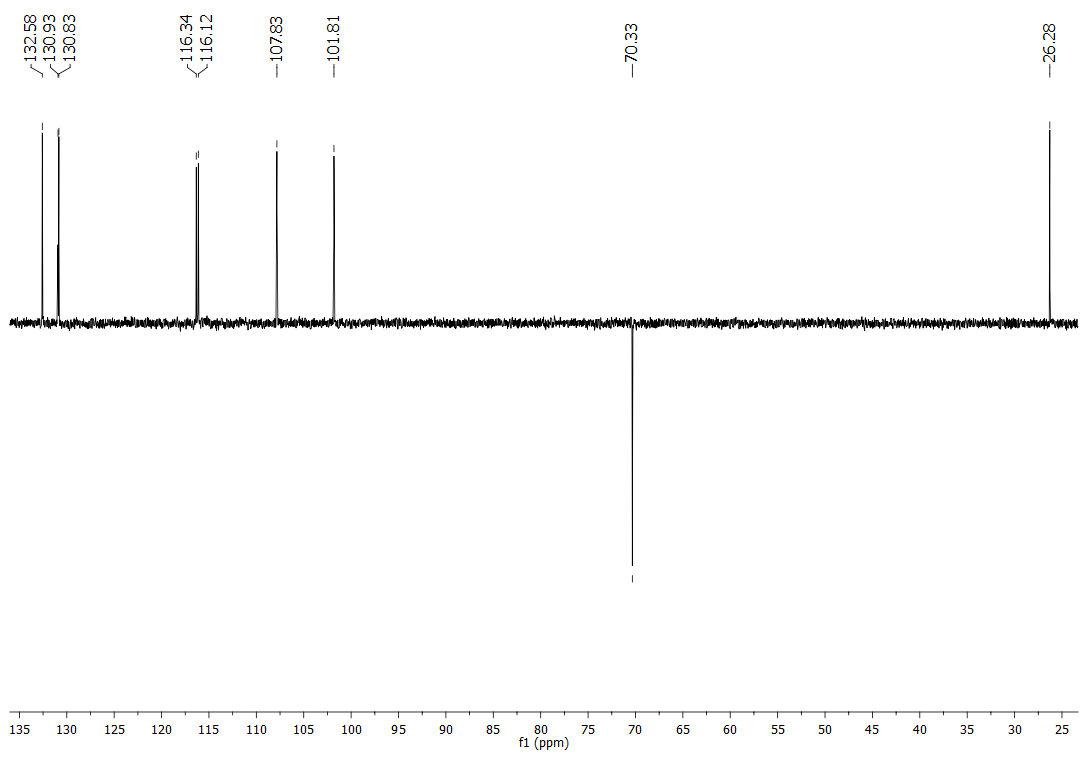


# ^1^H, ^13^C and DEPT NMR spectra of 3g

#


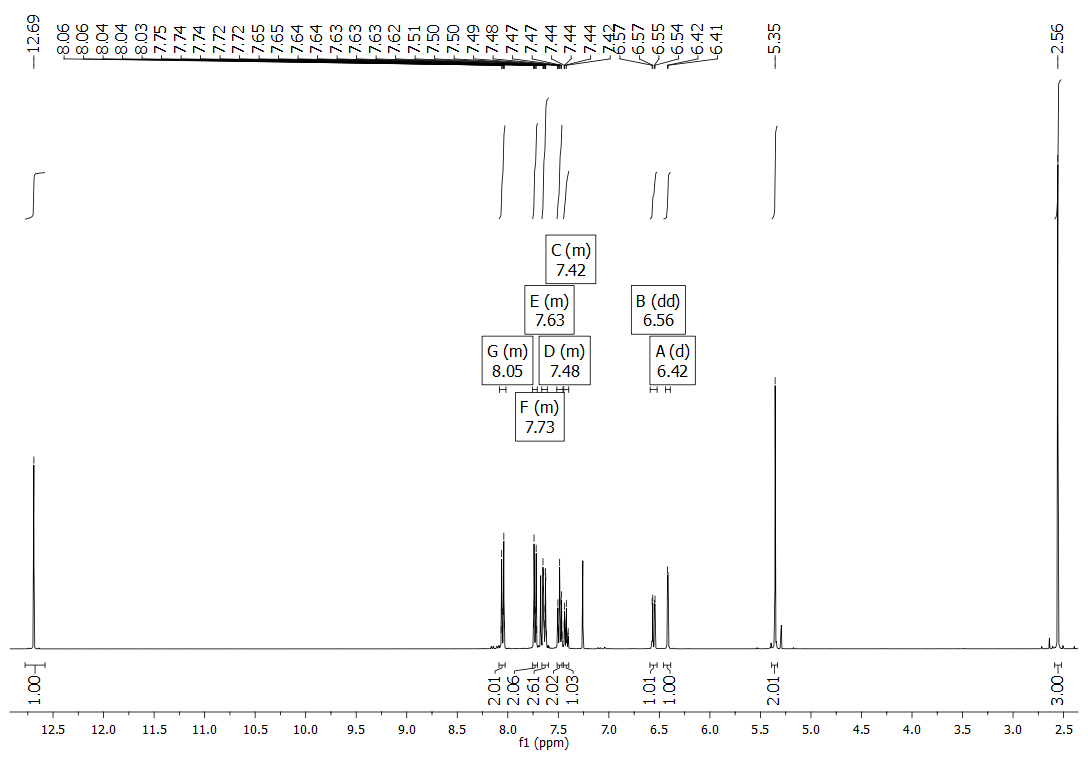


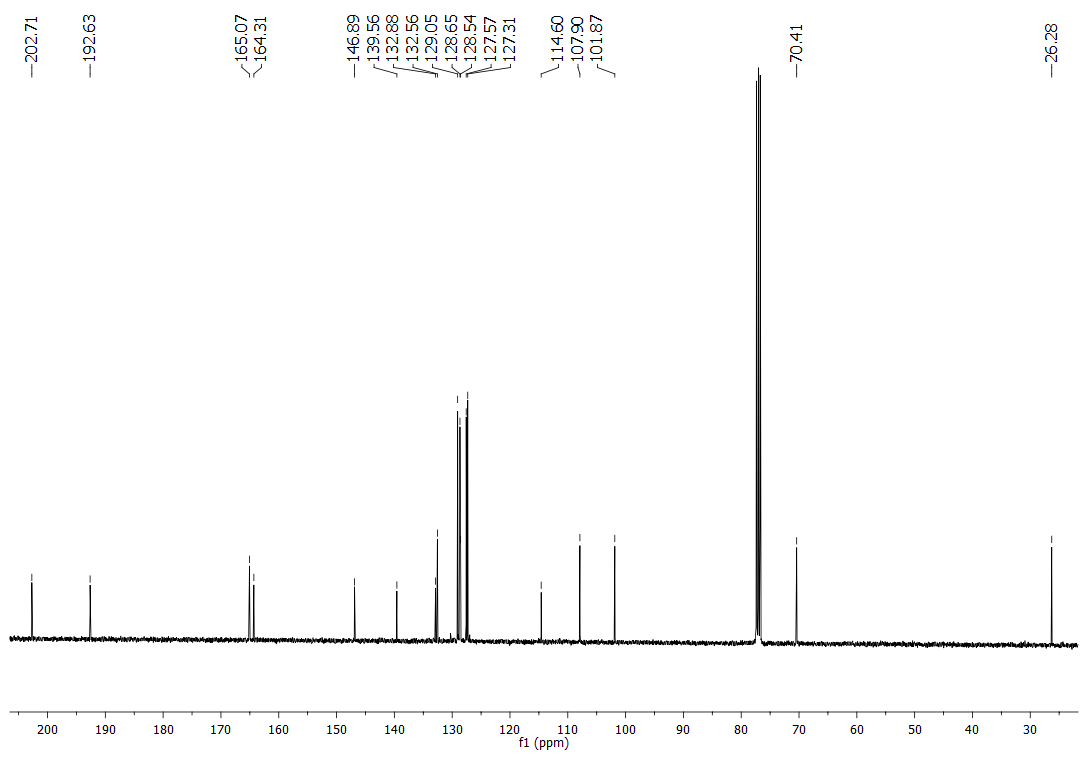


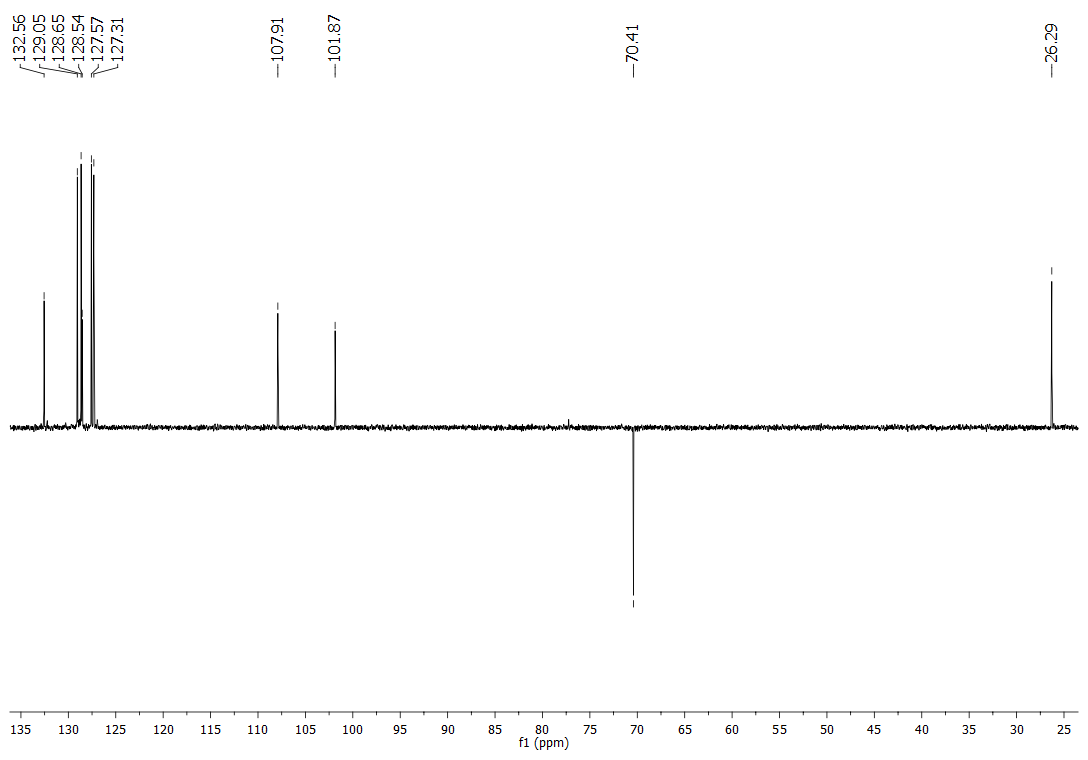


# ^1^H, ^13^C and DEPT NMR spectra of 3j


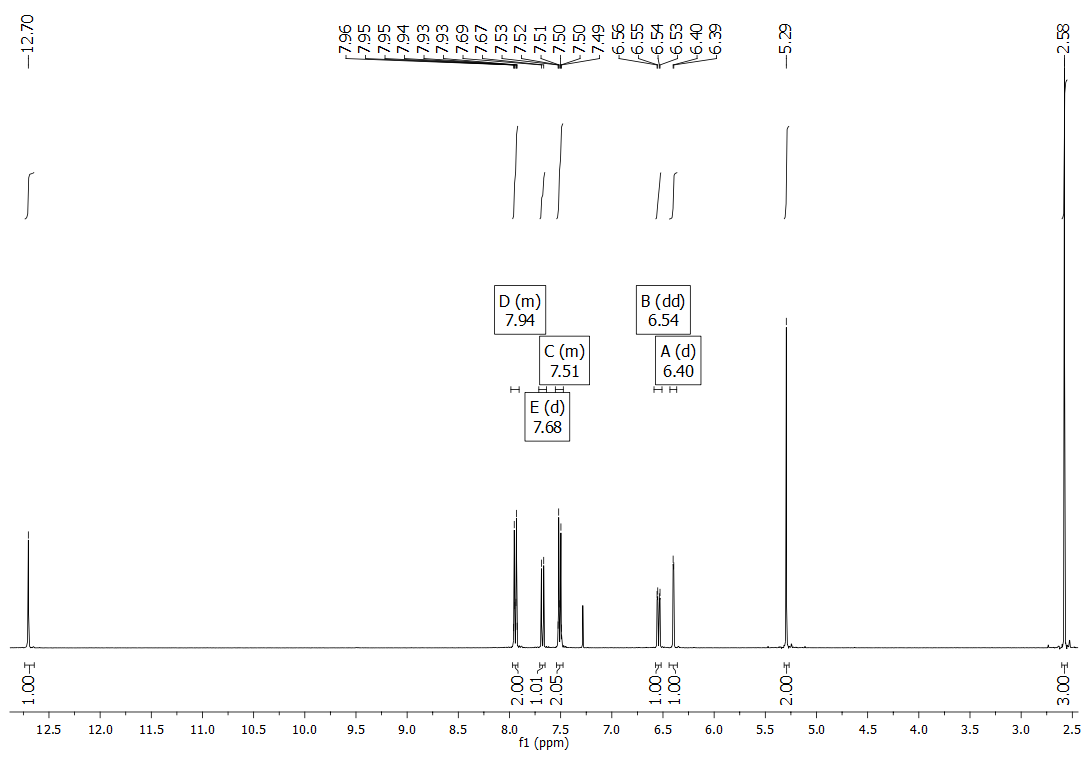


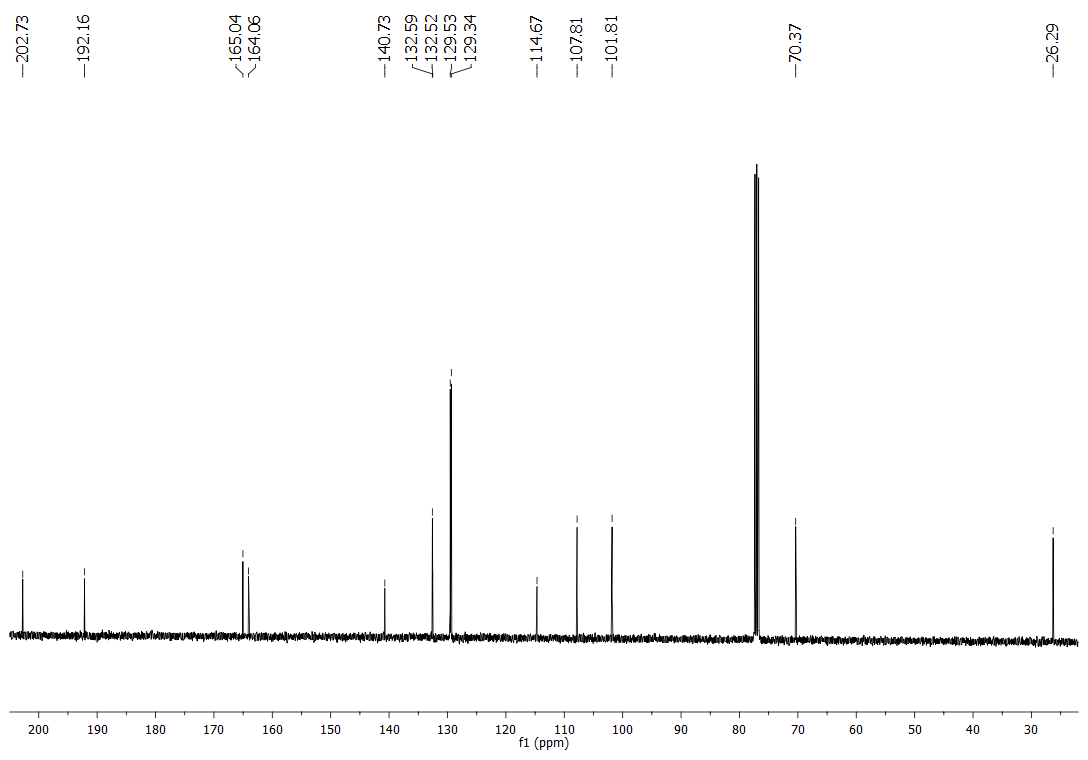


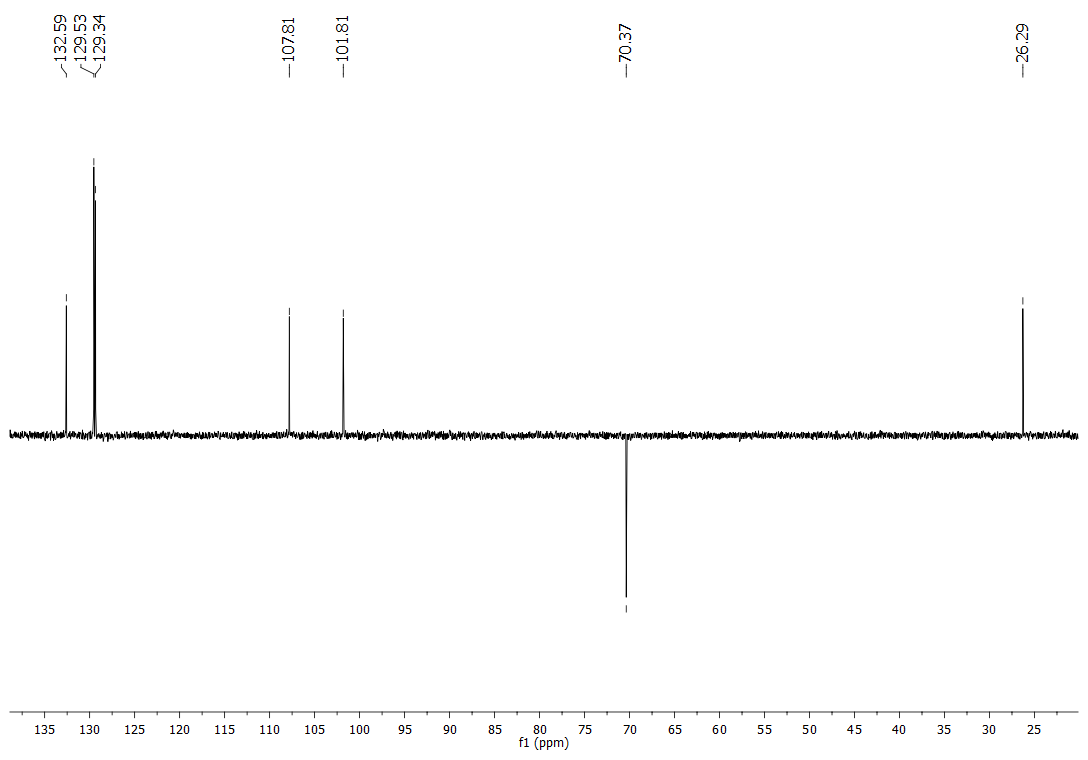


# ^1^H, ^13^C and DEPT NMR spectra of 3k


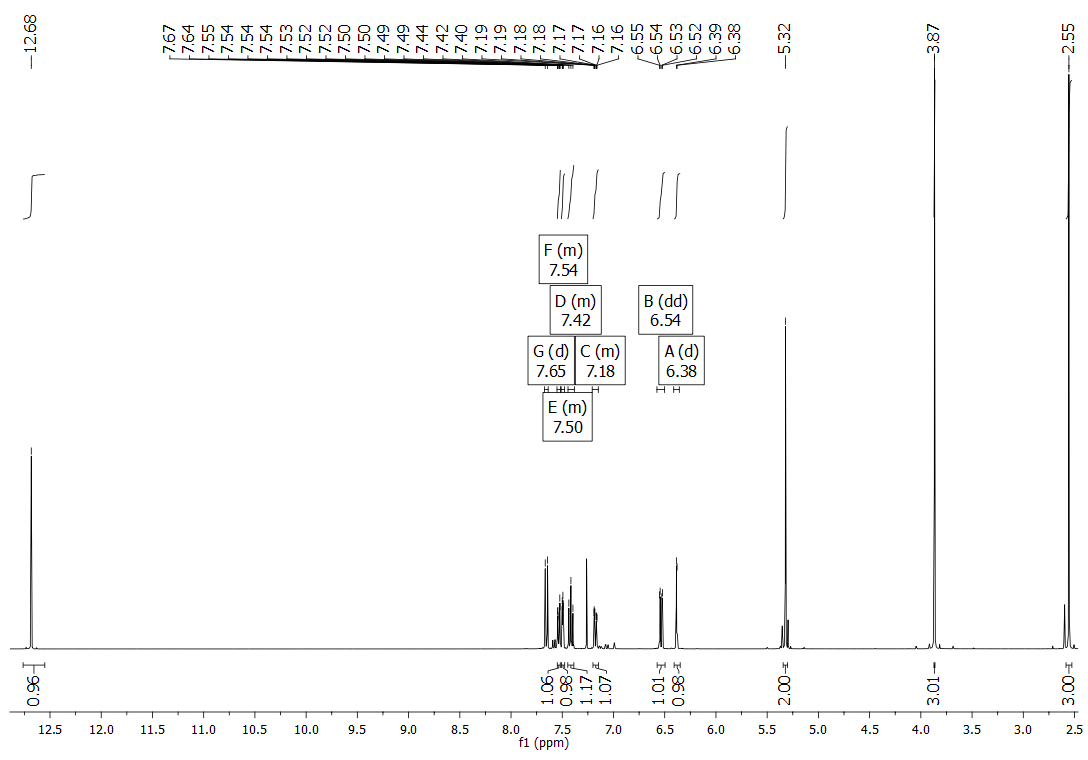


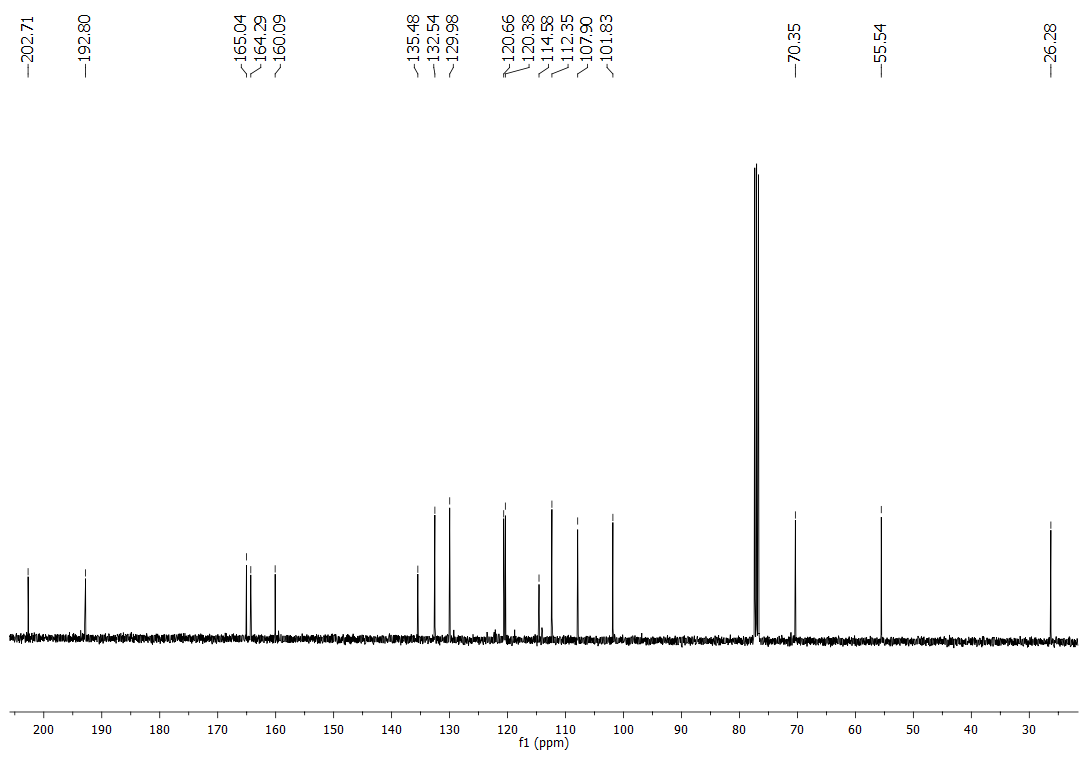


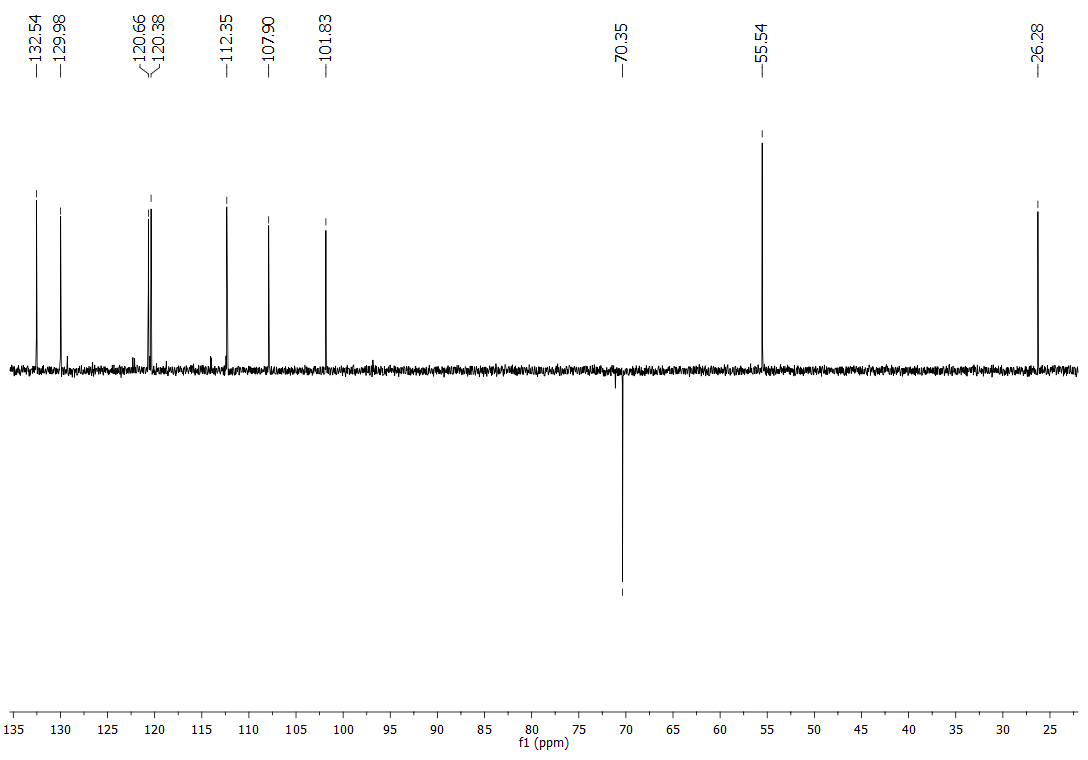


# ^1^H, ^13^C and DEPT NMR, and ESI/MS spectra, and chromatogram of 4a


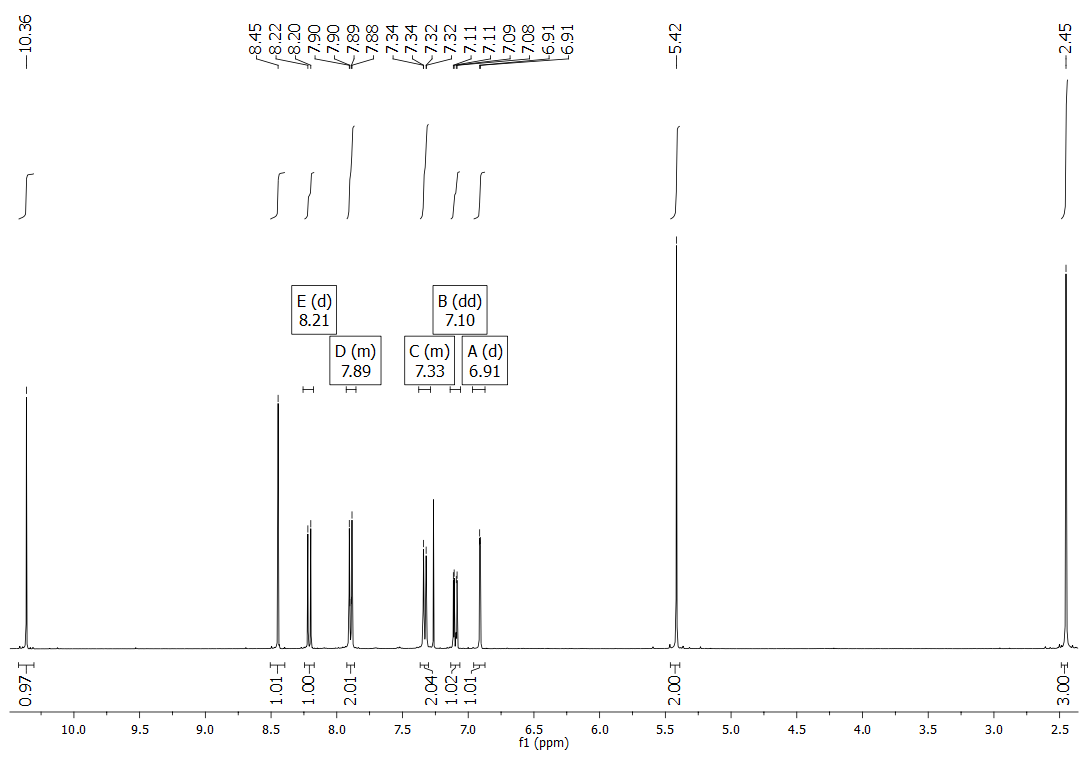


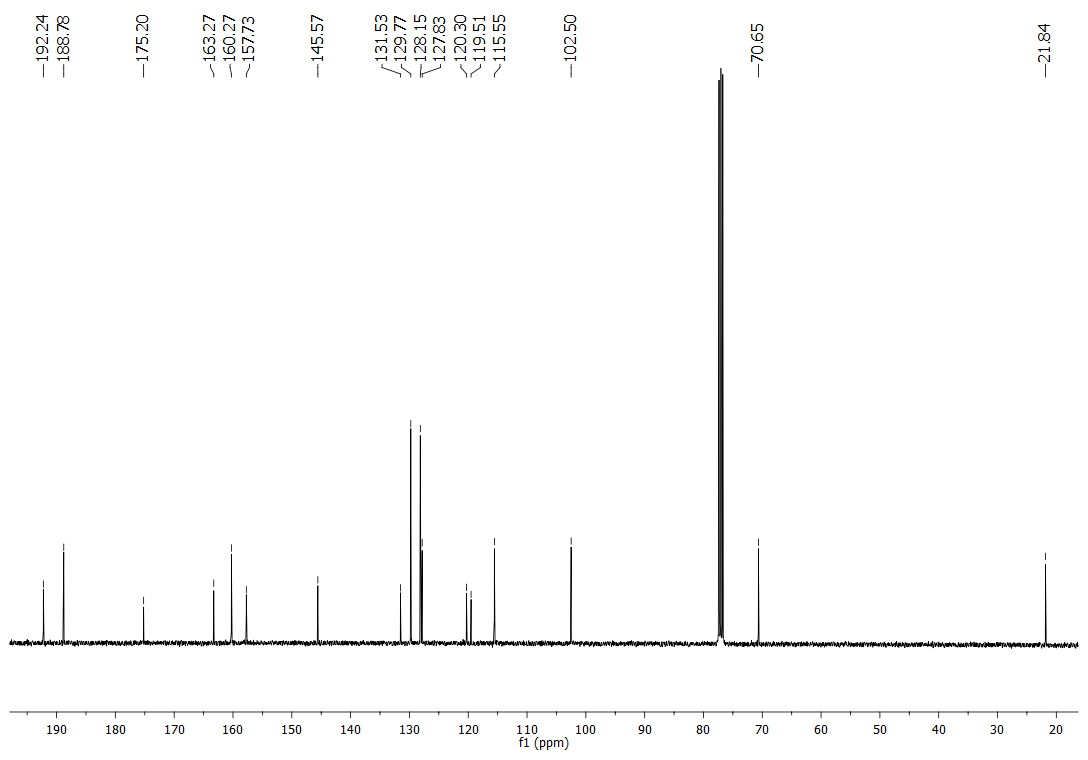


***
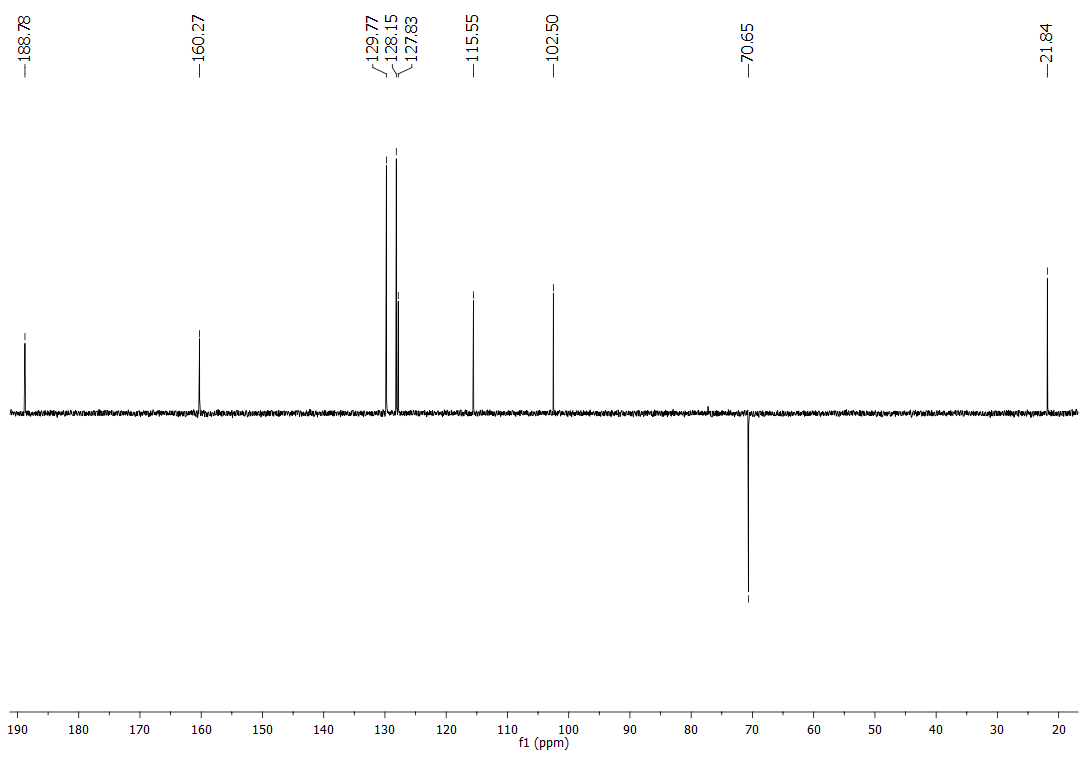
***

**
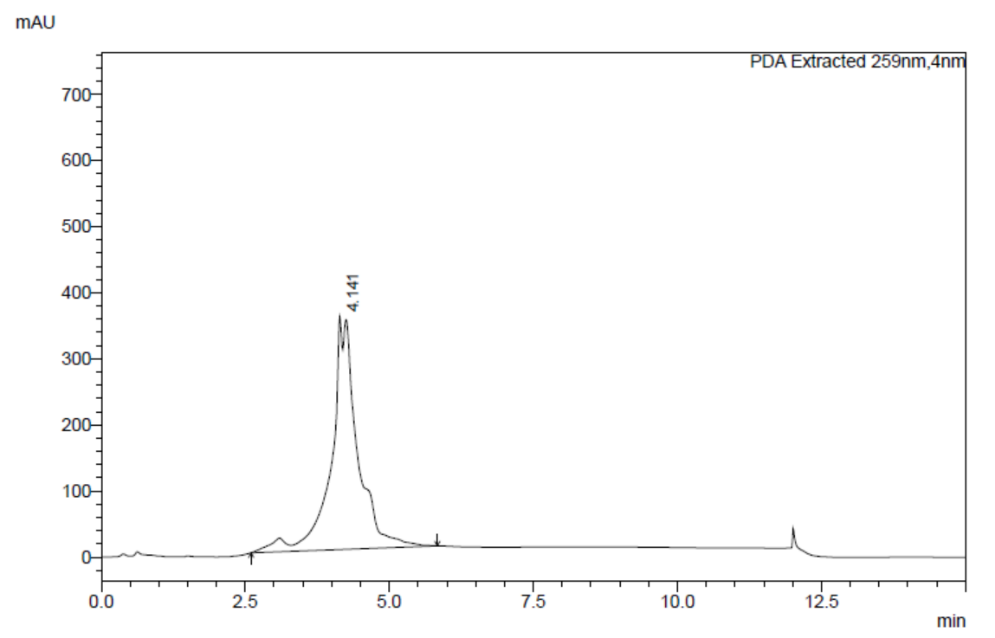
**

# ^1^H, ^13^C and DEPT NMR, and ESI/MS spectra, and chromatogram of 4b


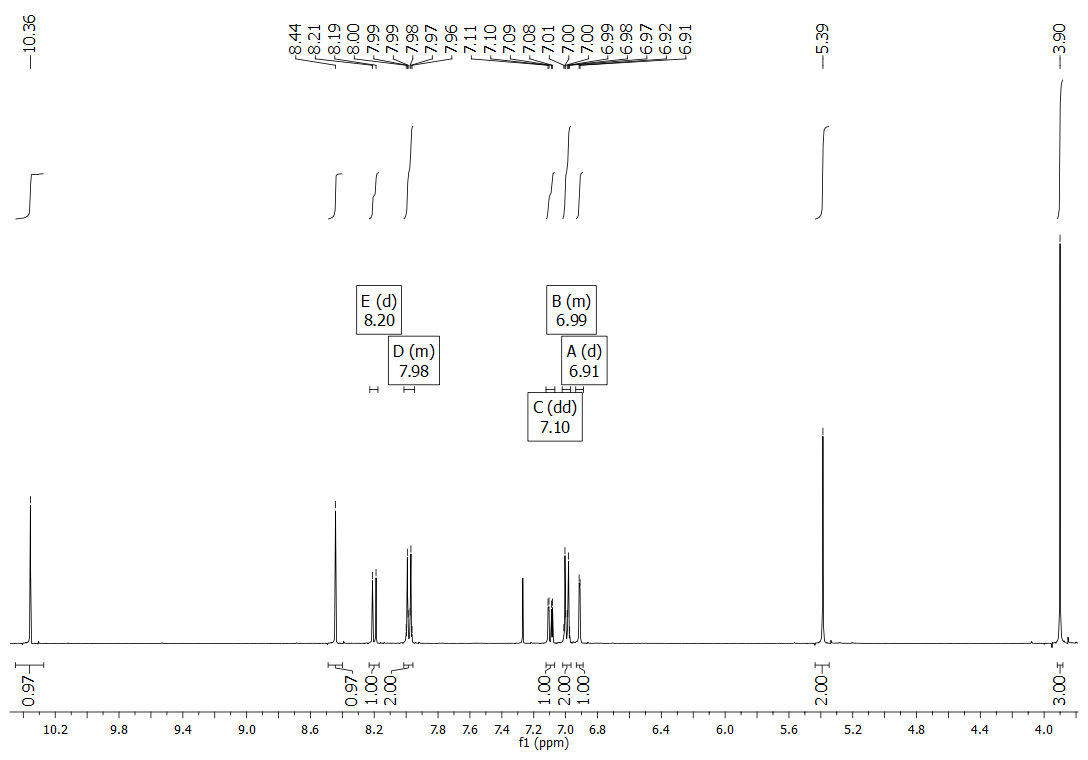


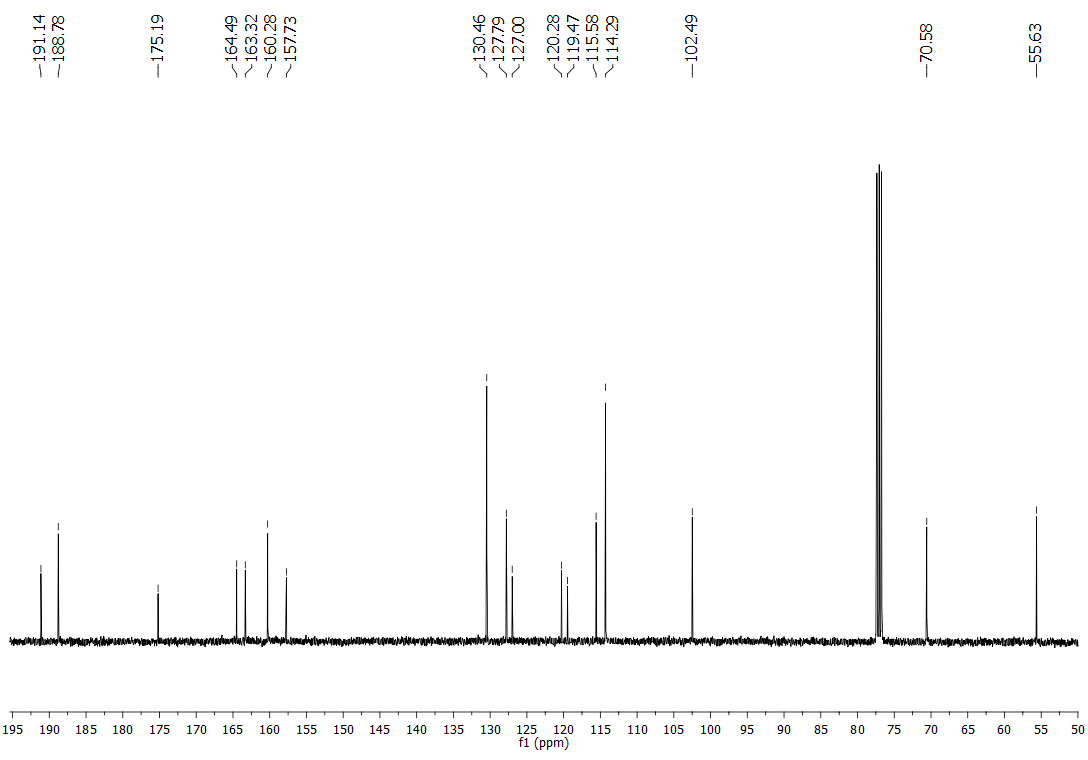


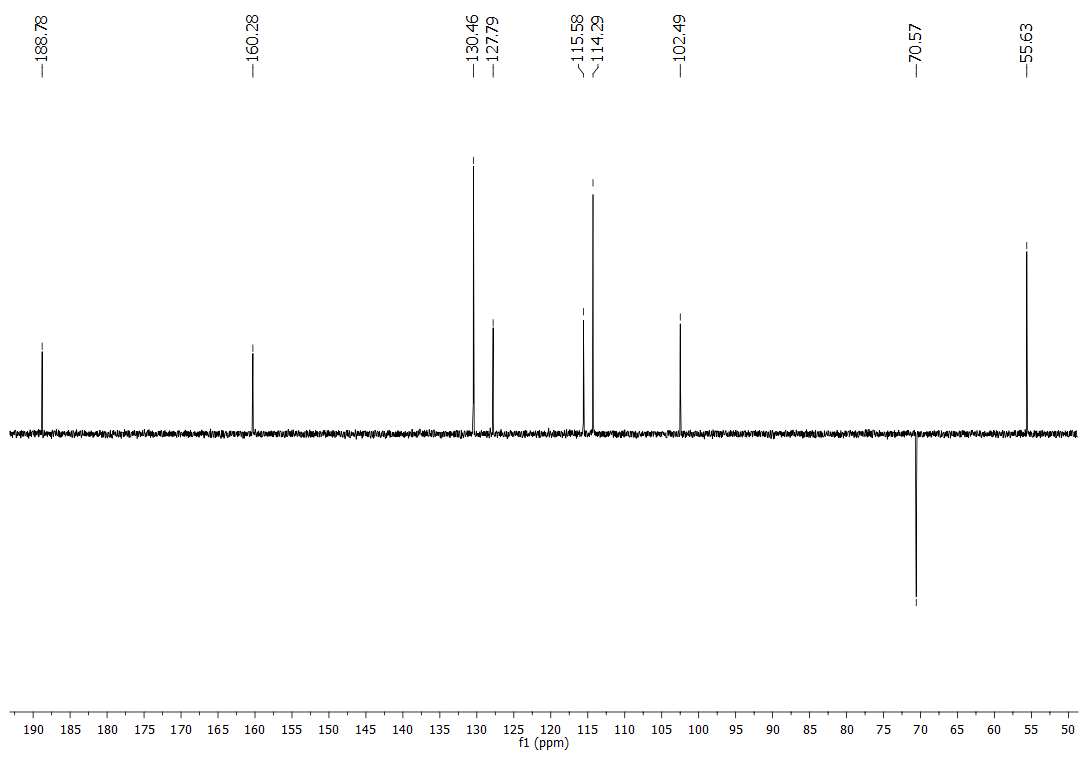


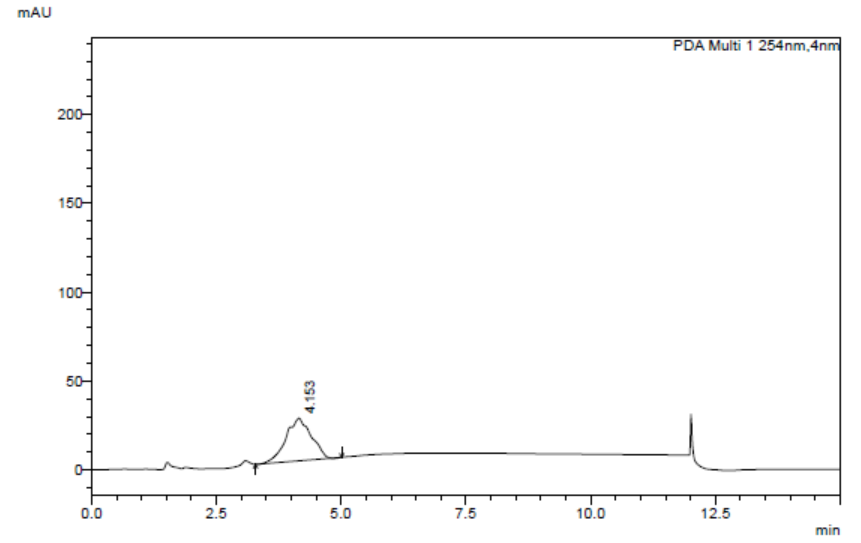


# ^1^H, ^13^C and DEPT NMR, and ESI/MS spectra, and chromatogram of 4c


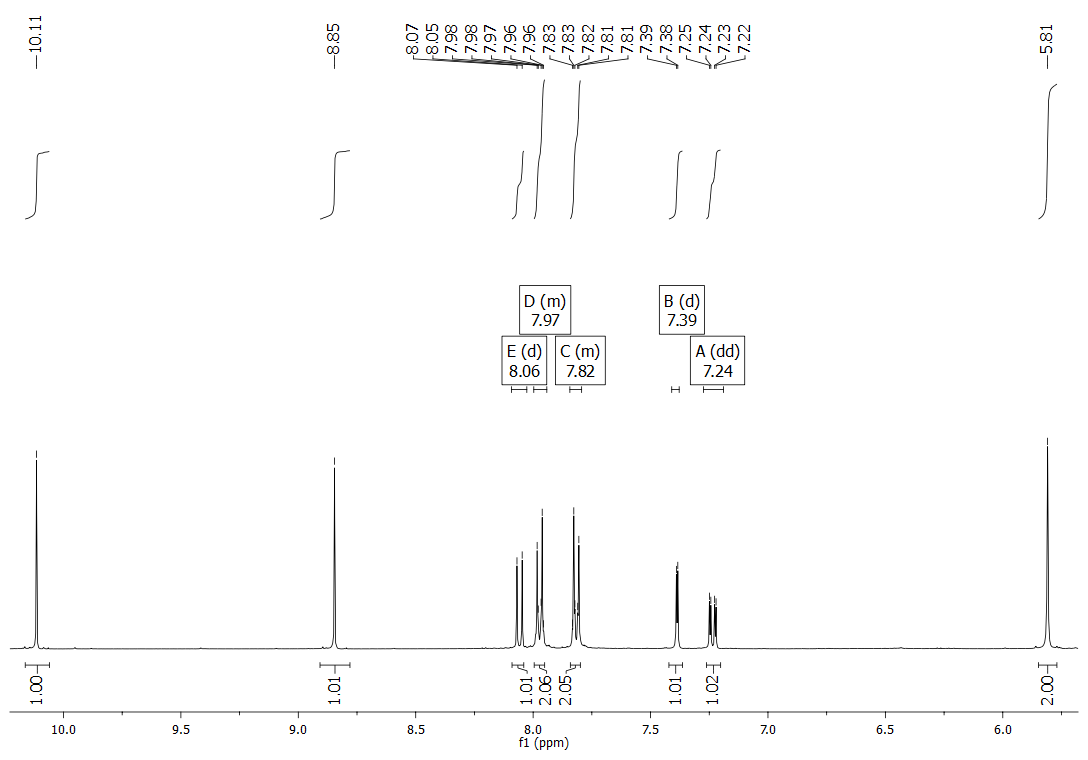


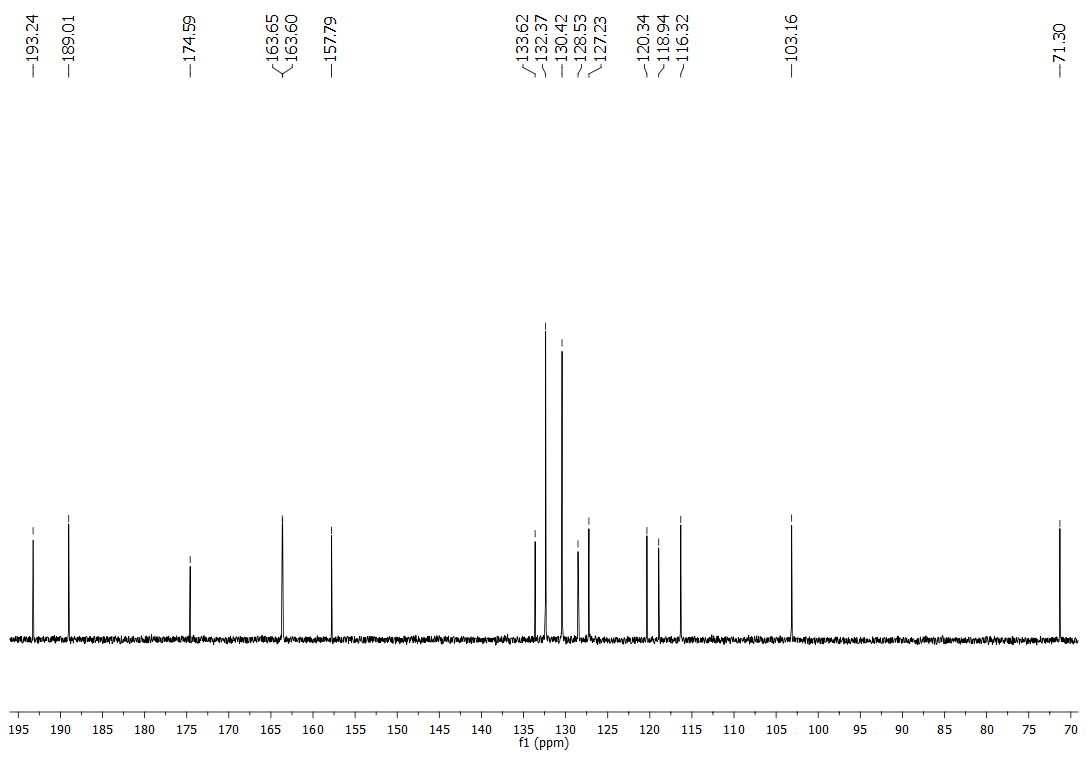


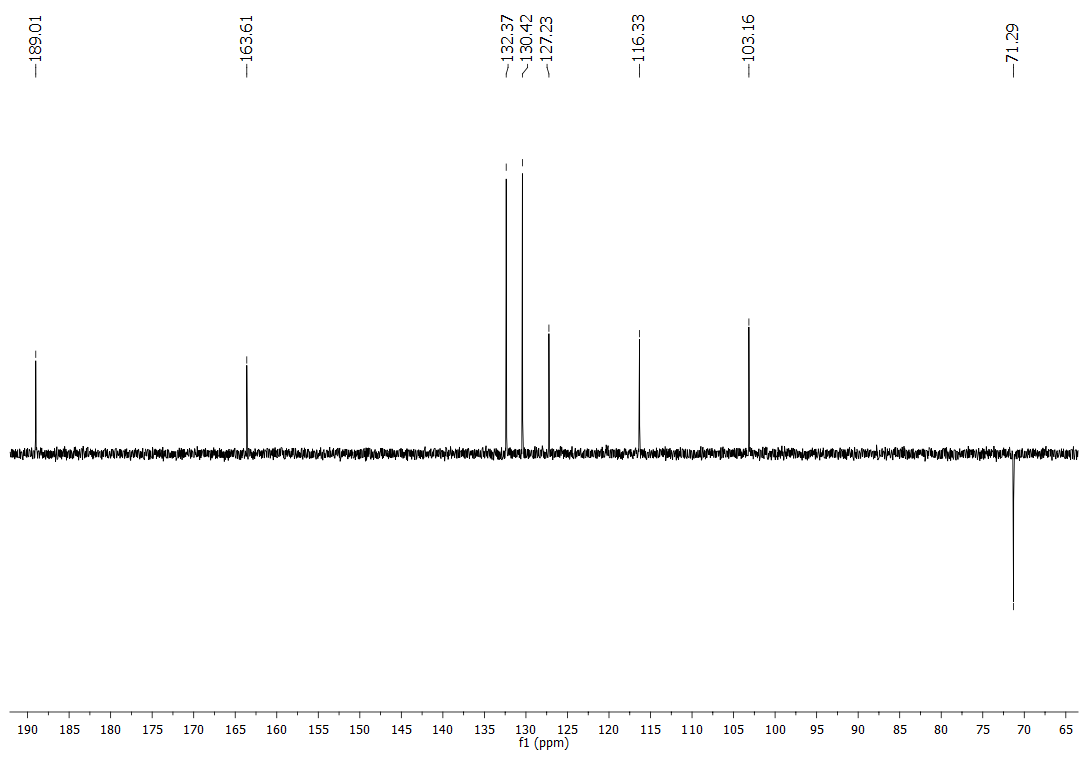


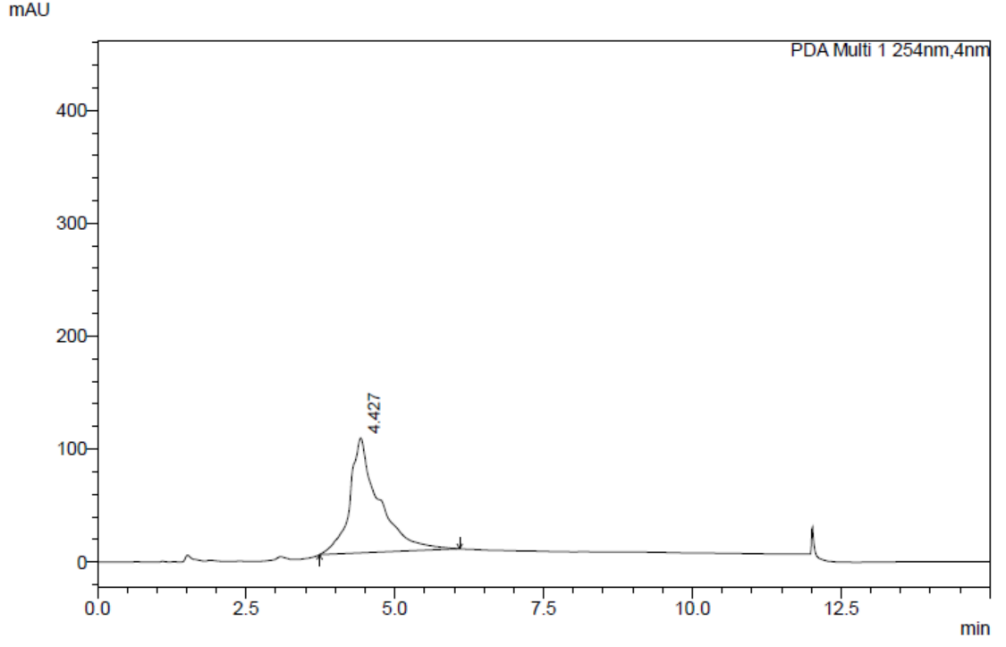


# ^1^H, ^13^C and DEPT NMR, and ESI/MS spectra, and chromatogram of 4d


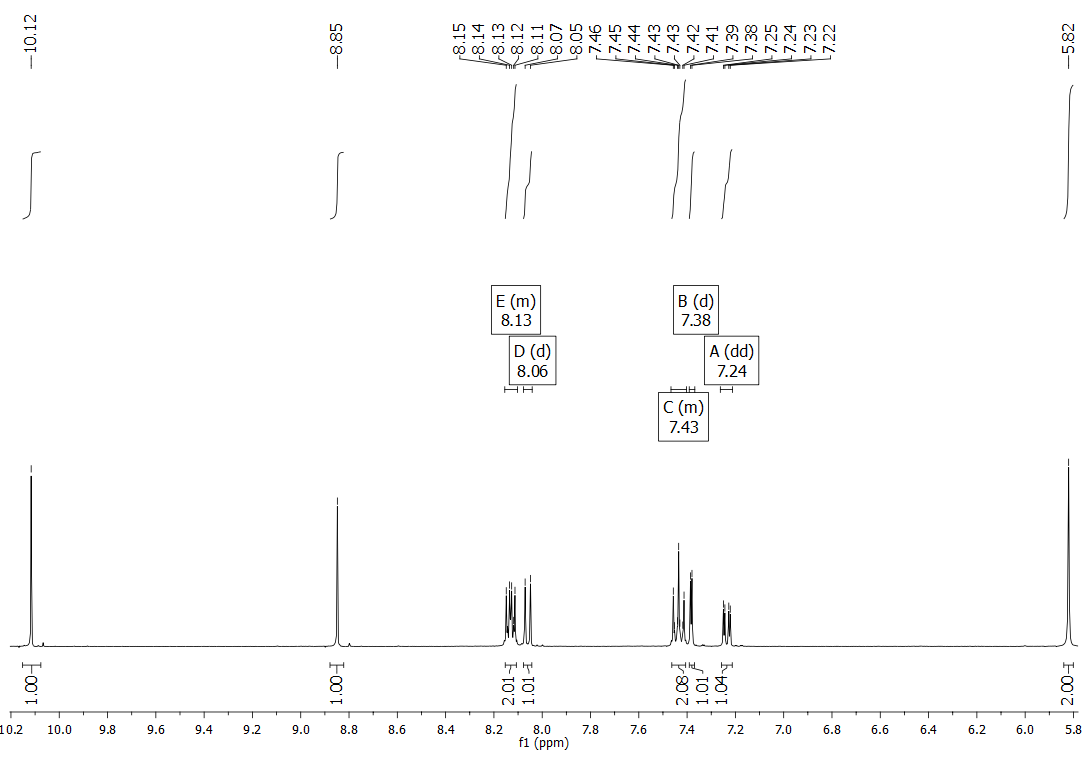


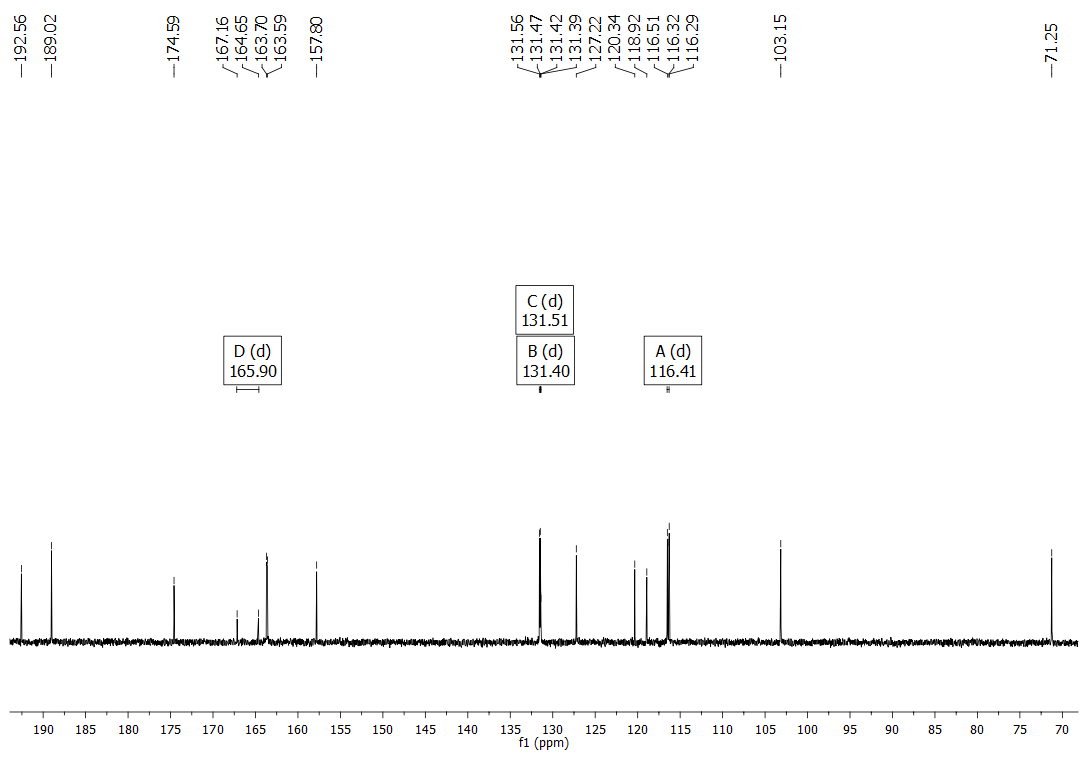


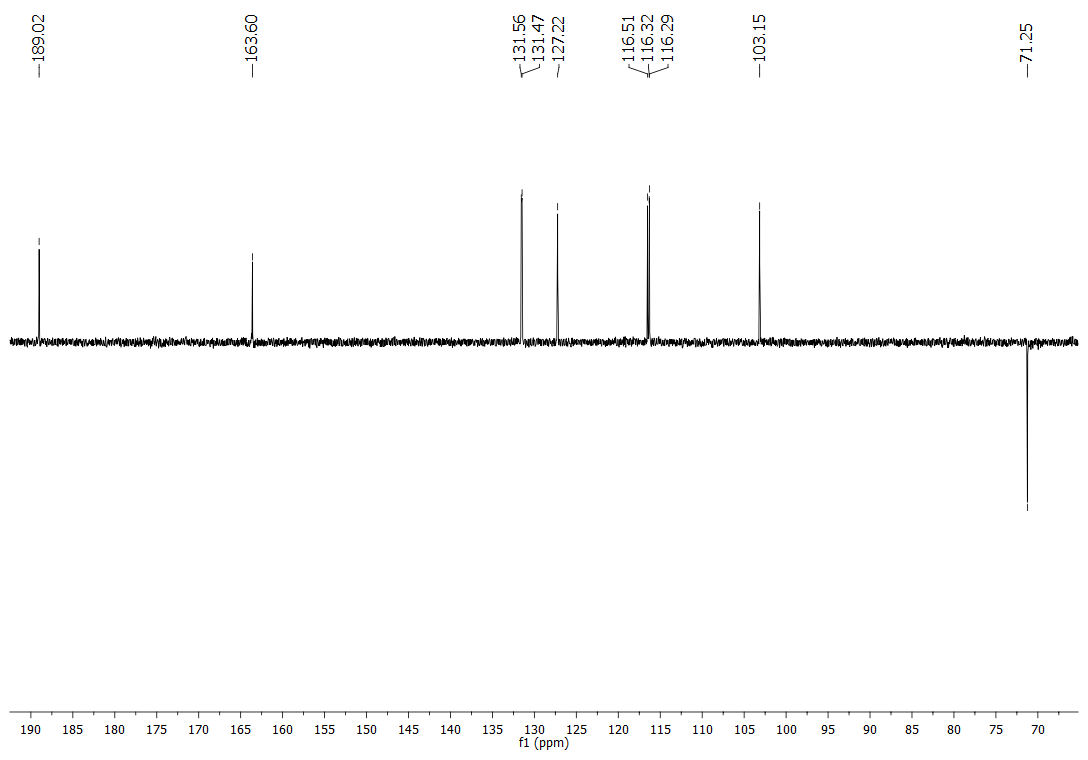


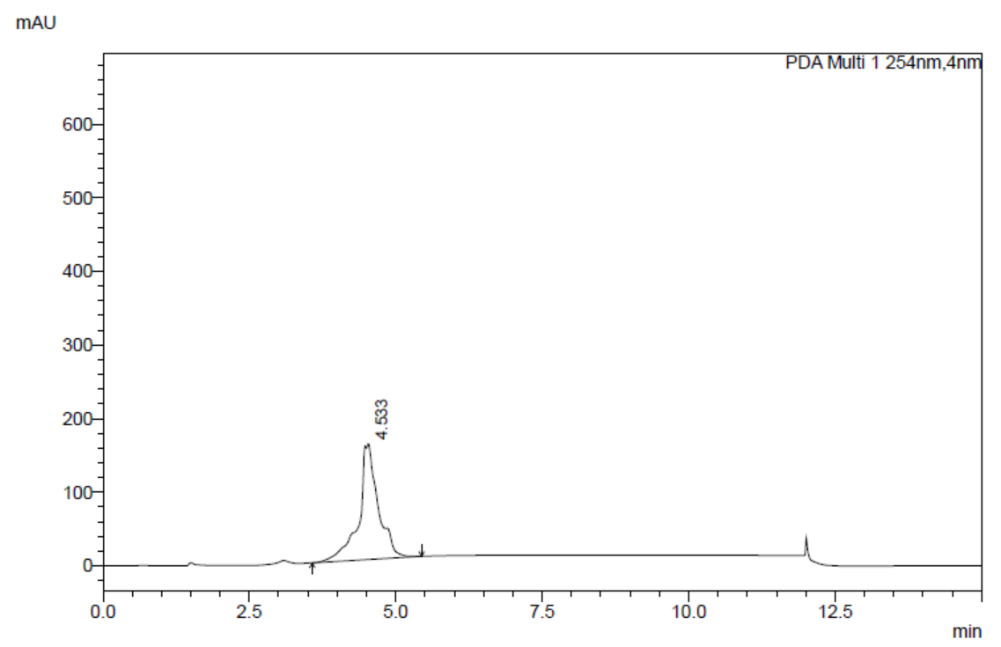


# ^1^H, ^13^C and DEPT NMR, and ESI/MS spectra, and chromatogram of 4g


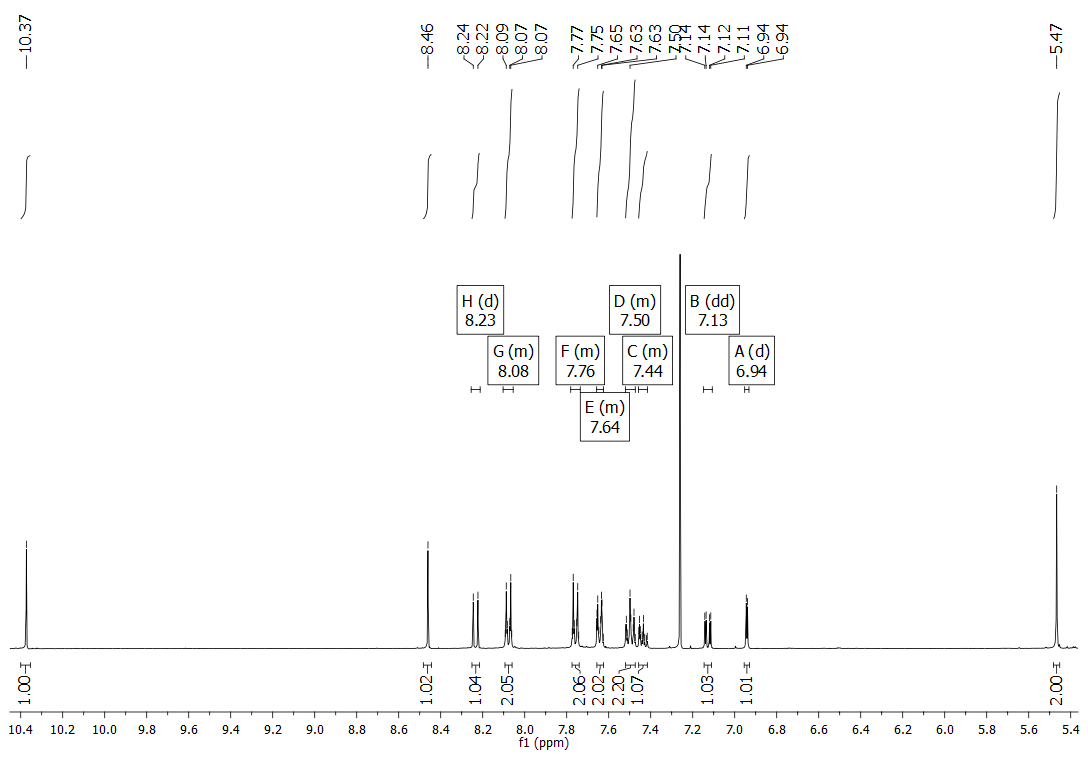


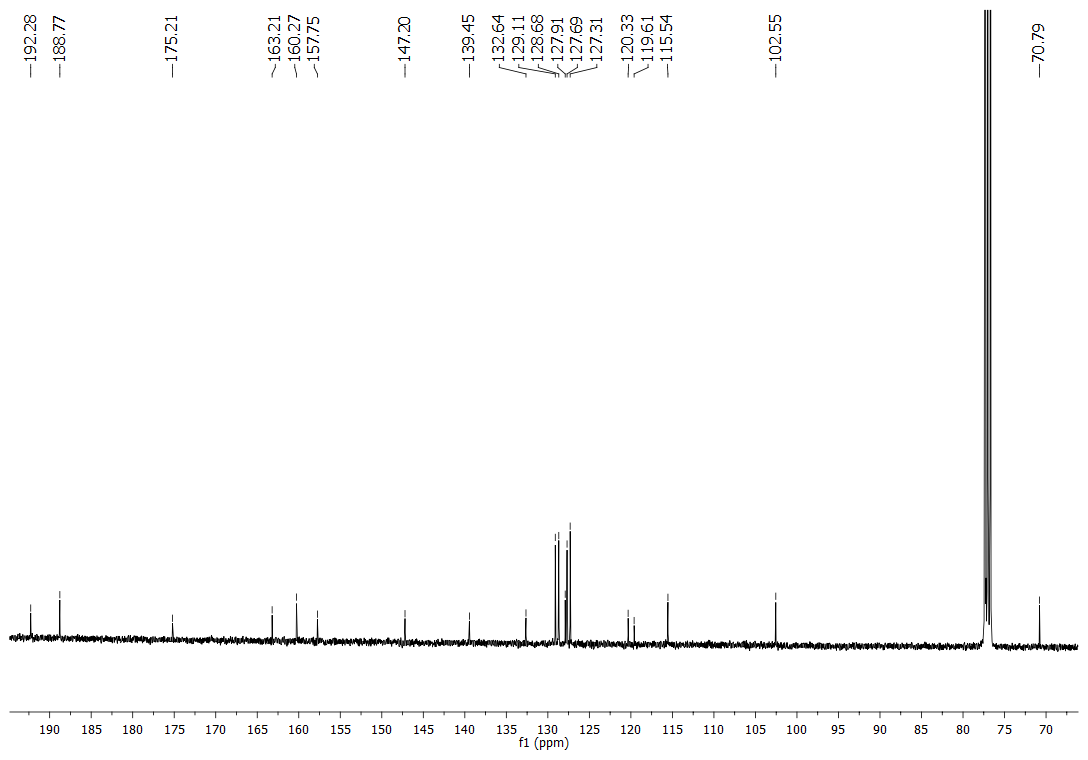


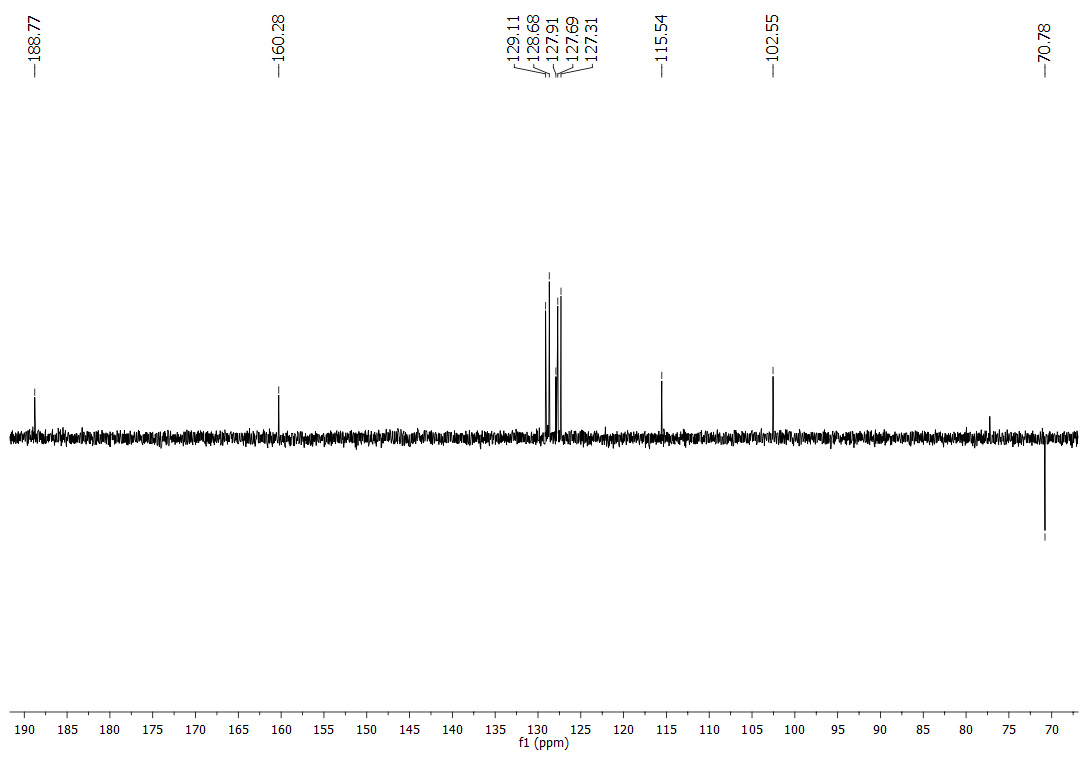


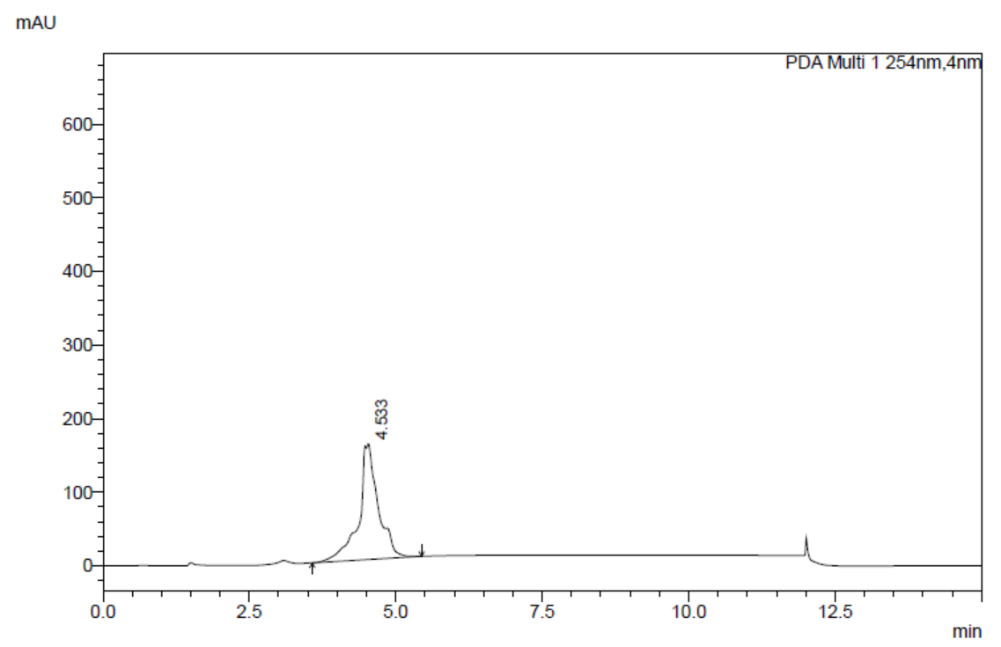


# ^1^H, ^13^C and DEPT NMR, and ESI/MS spectra of, and chromatogram 4j

***
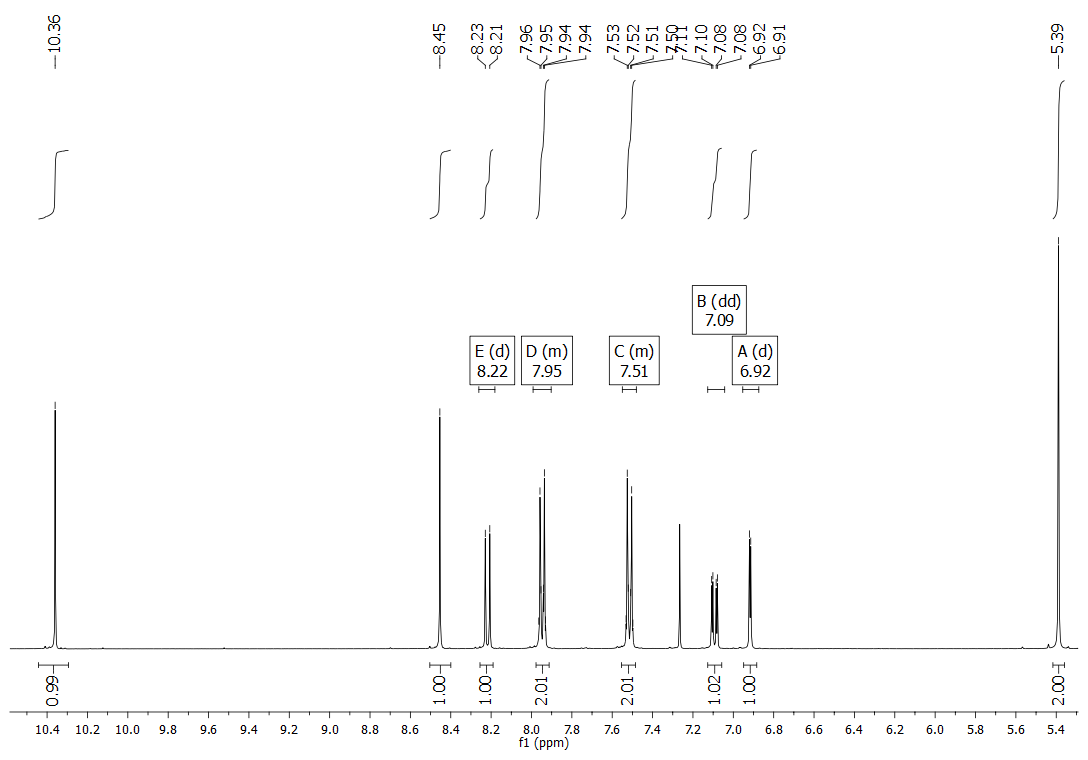
***

***
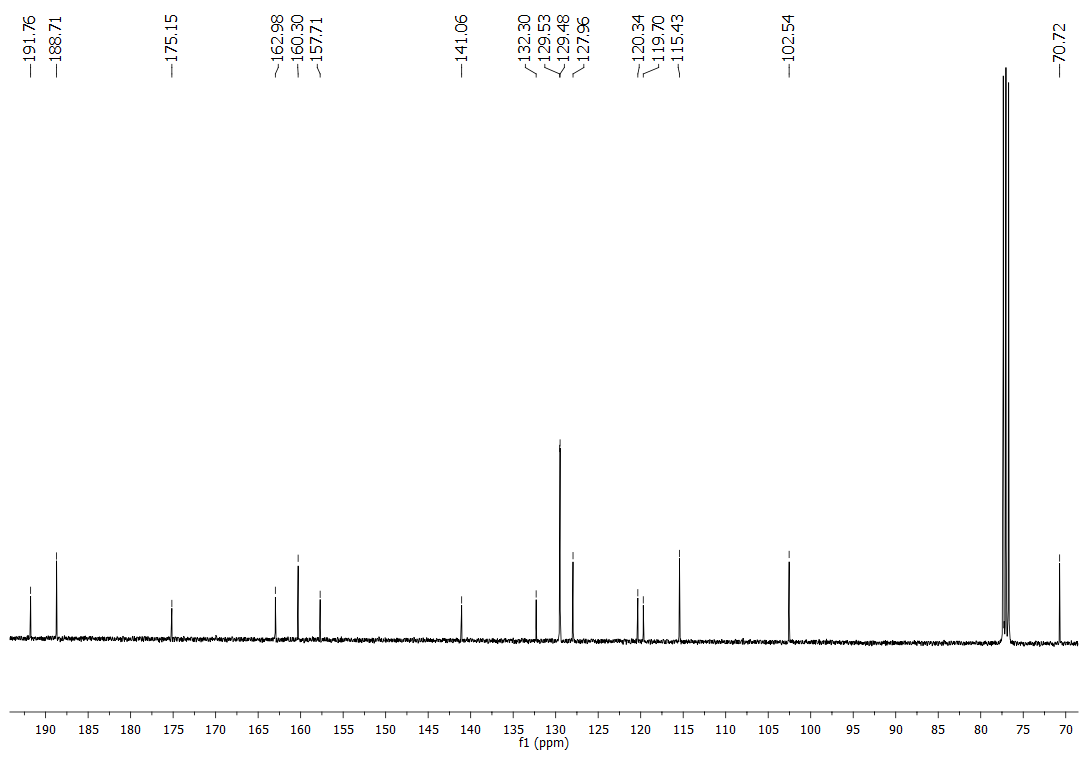
***

***
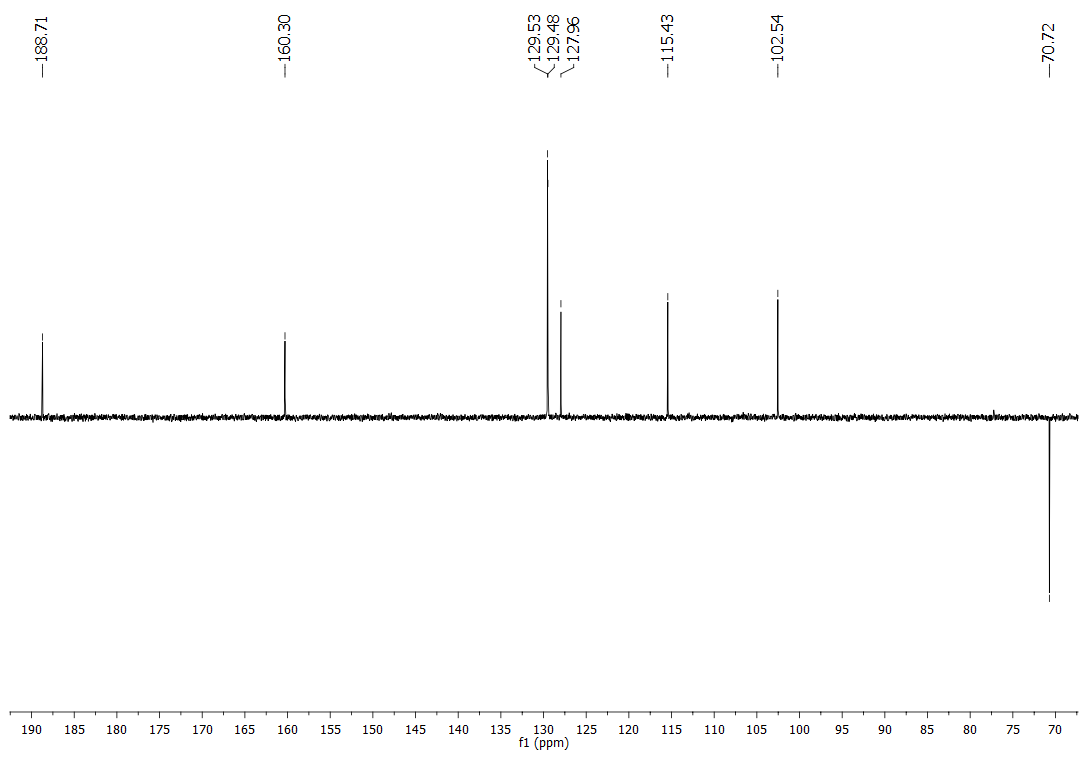
***


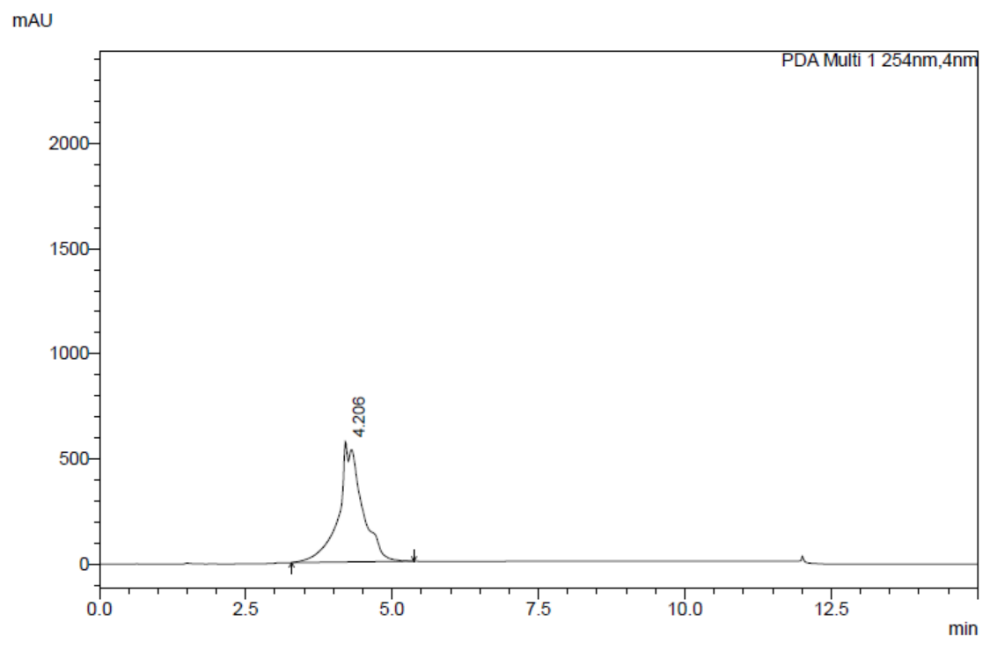


# ^1^H, ^13^C and DEPT NMR, and ESI/MS spectra, and chromatogram of 4k


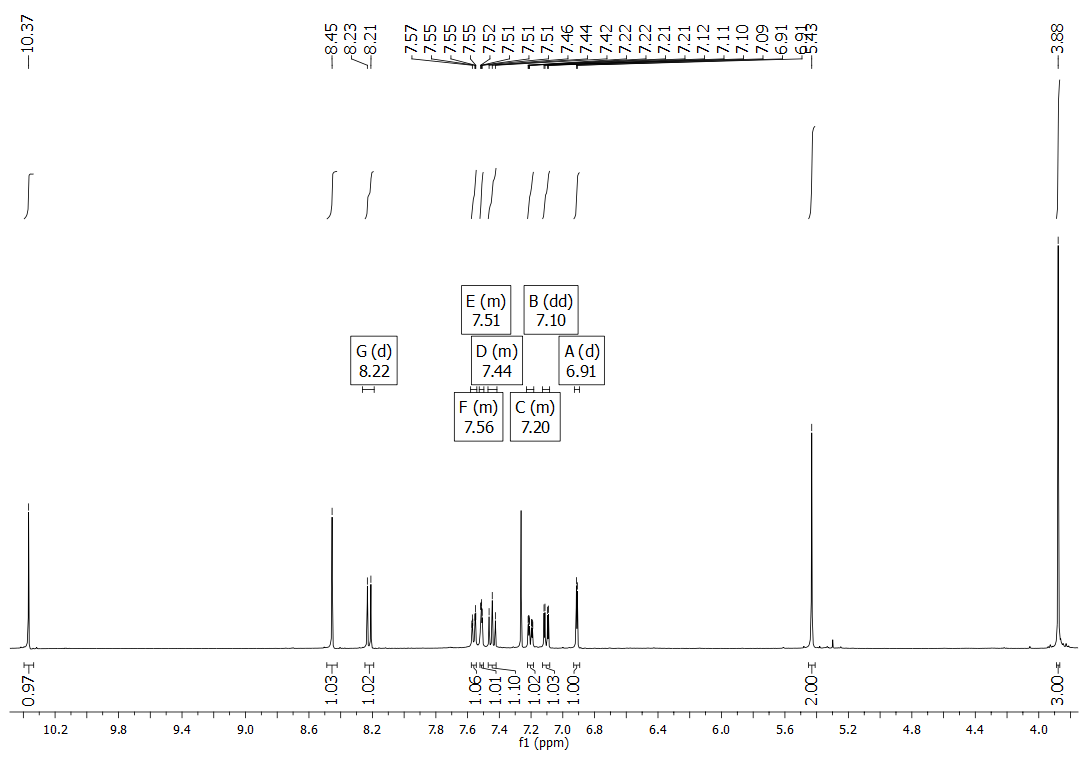


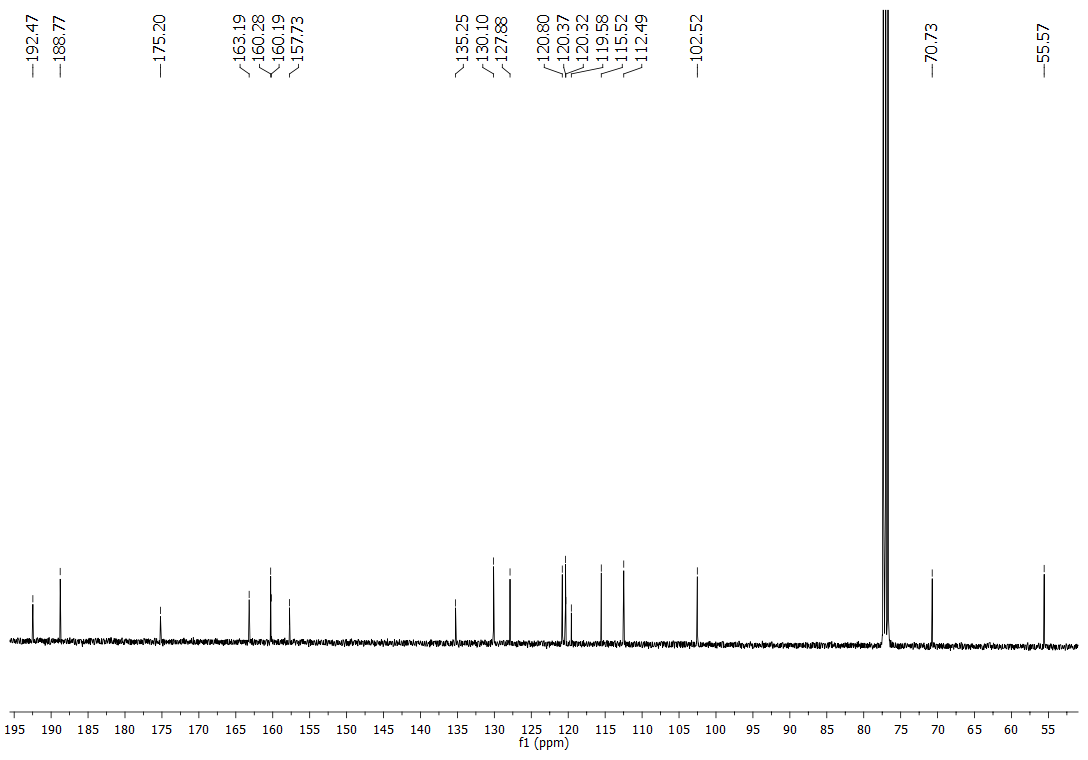


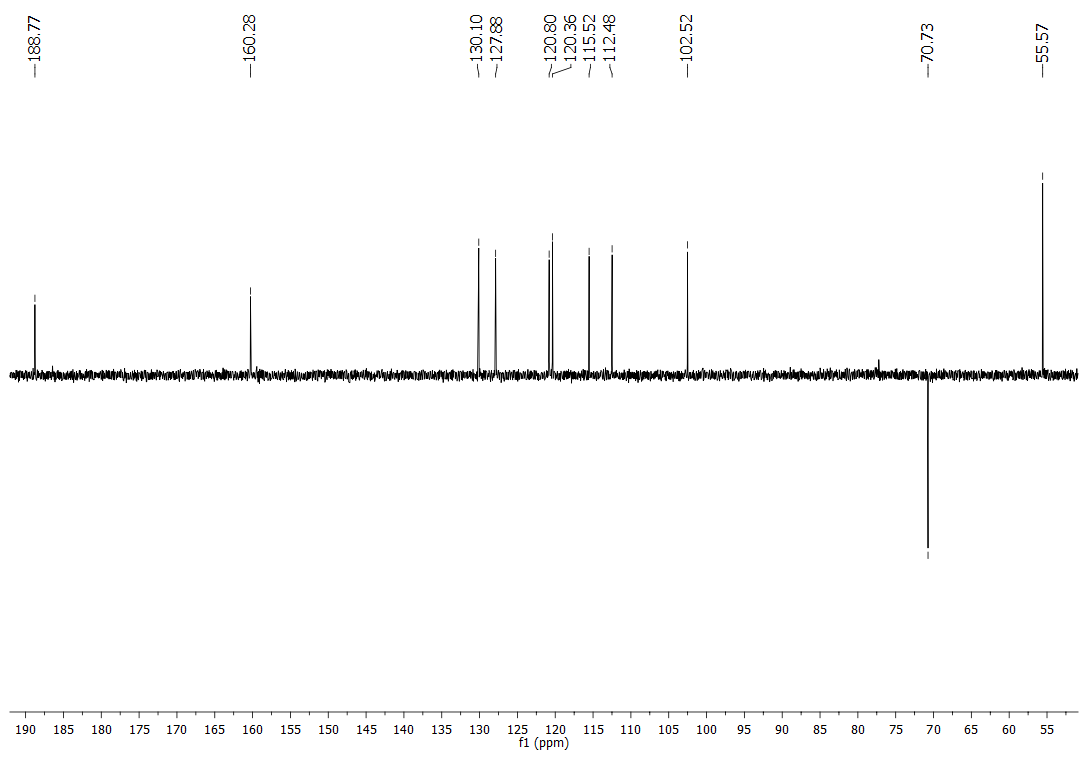


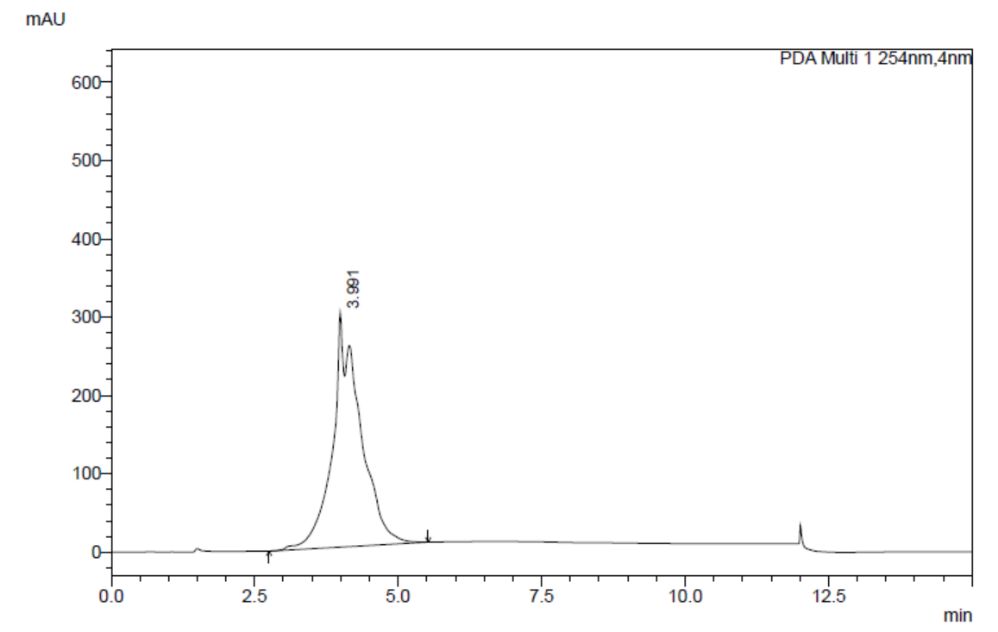


# Table S5. Calculated and experimental mass values for 4 series.

| **Compound** | **Molecular Formula** | **Mass** | **Theoretical Mass [M+H^+^]** | | **Experimental Mass [M+H^+^]** | | **Accuracy (ppm)** | |
| --- | --- | --- | --- | --- | --- | --- | --- | --- |
| **4a** | C_19_H_14_O_5_ | 322.32 | | 323.0920 | | 323.0912 | | -2.5 |
| **4b** | C_19_H_14_O_6_ | 338.32 | | 339.0868 | | 339.0860 | | -2.4 |
| **4c** | C_18_H_11_BrO_5_ | 387.19 | | 386.9868 | | 386.9861 | | -1.8 |
| **4d** | C_18_H_11_FO_5_ | 326.28 | | 327.0669 | | 327.0661 | | -2.4 |
| **4g** | C_24_H_16_O_5_ | 384.10 | | 385.1076 | | 385.1068 | | -2.1 |
| **4j** | C_18_H_11_ClO_5_ | 342.03 | | 343.0374 | | 343.0366 | | -2.3 |
| **4k** | C_19_H_14_O_6_ | 338.32 | | 339.0868 | | 339.0860 | | -2.4 |

# Table S6. Cross-docking results considering the co-crystallized ligands reported in pdb complexes. Receptor coordinates were obtained considering *h*CA IX pdb code 5FL4, and *h*CA XII pdb code 5MSA.

| ***h*CA IX** | | | ***h*CA XII** | | |
| --- | --- | --- | --- | --- | --- |
| **PDB** | **PDB Resolution (Å)** | **RMSD** | **PDB** | **PDB Resolution (Å)** | **RMSD** |
| **5FL4** | 1.82 | 1.73 | **5MSA** | 1.20 | 1.26 |
| **3IAI** | 2.20 | 2.00 | **4KP5** | 1.45 | 1.69 |
| **5FL5** | 2.05 | 1.84 | **6T5P** | 1.50 | 1.62 |
| **5FL6** | 1.95 | 1.84 | **1JD0** | 1.50 | 1.66 |
| **6FE0** | 1.91 | 1.97 | **5LLP** | 1.48 | 1.68 |
| **6FE1** | 1.95 | 1.93 | **6G5L** | 1.21 | 1.10 |
| **6G98** | 2.47 | 8.62 | **6QN0** | 1.89 | 2.43 |
| **6G9U** | 1.75 | 8.50 | **4Q0L** | 2.00 | 2.23 |
| **6QN2** | 1.95 | 1.93 | **4QJO** | 1.80 | 2.12 |
| **6QN6** | 2.25 | 1.01 | **4QJW** | 1.55 | 4.75 |
| **6TL5** | 2.21 | 5.92 | **5LL9** | 1.45 | 1.57 |
| **6TL6** | 2.15 | 1.72 | **6R71** | 2.00 | 0.85 |
|  |  |  | **5MSB** | 1.30 | 1.12 |
|  |  |  | **4KP8** | 1.80 | 4.60 |
|  |  |  | **4HT2** | 1.45 | 1.79 |
|  |  |  | **4WW8** | 1.42 | 2.03 |
|  |  |  | **5LLO** | 1.60 | 1.28 |
|  |  |  | **6QNG** | 1.67 | 1.54 |
|  |  |  | **6G7A** | 1.42 | 2.13 |
|  |  |  | **4QJ0** | 1.55 | 1.79 |
|  |  |  | **6R6Y** | 1.38 | 1.73 |
|  |  |  | **5LL5** | 1.42 | 2.52 |
|  |  |  | **5T5Q** | 1.95 | 1.55 |

# **Figure S1.** Calibration curve for CHI measurements using a mixture solution with 11 compounds with known CHI values.

# **Table S7.** Values obtained for the retention time of each compound present in the mixture solution for the measurements of CHI using a UHPLC system.

| **Pattern compounds** | **CHI_0_ pH 7.4** | **t_r_ mean (mixture)** |
| --- | --- | --- |
| Theophyline | 18.19 | 4.428 |
| Paracetamol | 20.59 | 4.567 |
| Caffeine | 24.12 | 4.726 |
| Benzimidazole | 30.71 | 5.101 |
| Colchicine | 44.32 | 5.567 |
| Carbamazepine | 58.45 | 6.281 |
| Indole | 69.15 | 6.887 |
| Propiophenone | 78.41 | 7.228 |
| Butyrophenone | 88.49 | 7.688 |
| Valerophenone | 97.67 | 8.093 |
| Heptanophenone | 111.8 | 8.799 |

# **Figure S2.** Calibration curve for CHI(IAM) measurements using a mixture solution with 9 compounds with known CHI(IAM) values.

# **Table S8.** Values obtained for the retention time of each compound present in the mixture solution for the measurements of CHI(IAM) using a UHPLC system.

| **Pattern compounds** | **CHI_(IAM)_ pH 7.4** | **t_r_ mean (mixture)** |
| --- | --- | --- |
| Paracetamol | 2.9 | 2.655 |
| Acetanilide | 11.57 | 3.519 |
| Acetophenone | 17.07 | 3.966 |
| Propiophenone | 26.39 | 4.684 |
| Butyrophenone | 32.55 | 5.238 |
| Valerophenone | 37.71 | 5.704 |
| Hexanophenone | 41.91 | 6.095 |
| Heptanophenone | 45.49 | 6.422 |
| Octanophenone | 49.40 | 6.710 |

# **Figure S3.** Calibration curve of Log K calculated by Log[HSA%/(101-HAS%)] and the logarithmic of retention time of a mixture of compounds with known percentage of binding with HSA.

# **Table S9.** Values of retention time obtained for the compounds used in the mixture solution and the values of Log K and Log (tr) calculated.

| **Pattern compounds** | **% HSA** | **Log K** | **tr (min)** | **Log tr** |
| --- | --- | --- | --- | --- |
| Caffeine | 14.34 | -0.78 | 2.07 | 0.32 |
| Lignocaine | 29.8 | -0.38 | 2.55 | 0.41 |
| Trimethoprim | 39.5 | -0.19 | 3.15 | 0.50 |
| Corticosterone | 67.8 | 0.31 | 4.12 | 0.61 |
| Propanolol | 74.25 | 0.44 | 4.47 | 0.65 |
| Testosterona | 87.9 | 0.83 | 4.96 | 0.70 |
| Piroxicam | 97.7 | 1.47 | 8.83 | 0.95 |
| Pyrene | 98.9 | 1.67 | 10.36 | 1.02 |
